# Strong Internal Electric Field‐Driven S‐scheme CoAl‐LDH/ZrO_2_ Heterojunction for Enhanced Photocatalytic CO_2_ Reduction: Configuration, Performance, and Mechanism

**DOI:** 10.1002/advs.202510939

**Published:** 2025-09-12

**Authors:** Mengwei Chen, Jiaze Xiao, Yongxin Lei, Xupeng Qin, Santosh K. Tiwari, Nannan Wang, Zhiyao Wu, Yanqiu Zhu, Xinpeng Wang

**Affiliations:** ^1^ State Key Laboratory of Featured Metal Materials and Life‐cycle Safety for Composite Structures Guangxi Key Laboratory of Processing for Non‐Ferrous Metals and Featured Materials School of Resources Environment and Materials Guangxi University Nanning 530004 China; ^2^ State Key Laboratory of Chemistry for NBC Hazards Protection Frontiers Science Center for Rare Isotopes School of Nuclear Science and Technology Lanzhou University Lanzhou 730000 China; ^3^ National Synchrotron Radiation Laboratory University of Science and Technology of China Hefei 230029 China; ^4^ Department of Chemistry NMAM Institute of Technology Karkala Karnataka 574110 India; ^5^ Faculty of Environment Science and Economy University of Exeter Exeter EX4 4QF UK

**Keywords:** CO_2_, internal electric field, layered double hydroxides, photocatalysis, S‐scheme heterojunction

## Abstract

The construction of S‐scheme heterojunctions with a strong internal electric field (IEF) is critical for enhancing photocatalytic performance. Herein, an S‐scheme heterojunction composed of CoAl‐LDH and ZrO_2_ (denoted as LZ‐60) is synthesized via a hydrothermal method. Under simulated solar irradiation, LZ‐60 exhibited a CO production rate of 562.545 µmol g^−1^ h^−1^, which is five times higher than pristine CoAl‐LDH and 43 times higher than pristine ZrO_2_. X‐ray photoelectron spectroscopy (XPS) revealed electron transfer from CoAl‐LDH to ZrO_2_ upon hybridization, generating an IEF at the interface. This electron transfer and IEF are further verified by density‐functional theory (DFT) calculations of work functions. Comparative XPS analysis before and after the photocatalytic reaction confirmed the S‐scheme charge transfer mechanism: the binding energies of Co and Al decreased, while Zr increased, indicating electron transfer from ZrO_2_ to CoAl‐LDH under light. Photoelectrochemical characterizations (PL, EIS) demonstrated enhanced charge separation in the heterojunction. In‐situ Fourier transform infrared spectroscopy identified CO* as the dominant intermediate, confirming high CO selectivity. The accelerated charge separation and strengthened redox capability synergistically contribute to the superior CO_2_ reduction performance of the S‐scheme LZ‐60 heterojunction. This work provides a valuable reference for designing efficient CO_2_ reduction photocatalysts.

## Graphical abstract

This study constructed an S‐scheme heterojunction catalyst CoAl‐LDH/ZrO_2_ driven by an internal electric field. The S‐scheme heterojunction accelerated charge separation and strengthened redox capability synergistically contribute to the superior CO_2_ reduction performance.Under simulated solar irradiation, optimal catalyst exhibited a CO production rate of 562.545 µmol g^−1^ h^−1^, which is 5 times higher than CoAl‐LDH and 43 times higher than ZrO_2_.

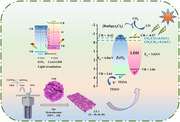

## 1 Introduction

As traditional fossil fuels deplete and the environmental crisis caused by carbon emissions grows increasingly severe, humanity is in urgent need of clean, renewable energy alternatives. In this context, photocatalytic technology has emerged as a transformative solution by mimicking natural photosynthesis to convert carbon dioxide into high‐value‐added fuels such as methanol and methane using solar energy,^[^ ^1^ ^]^ it can be traced back to 1911, and photocatalysis began to glow.^[^ ^2^ ^]^ This not only addresses environmental challenges but also accelerates the development of a clean, stable, and economically sustainable integrated energy value chain.^[^ ^3^ ^]^ Nowadays, photocatalytic carbon dioxide reduction reaction (CO_2_RR) research is rapidly becoming an important frontier in the fields of energy science and environmental engineering.

Despite remarkable advancements in solar‐driven photocatalytic CO_2_ reduction research, the development of photocatalysts for efficient CO_2_RR remain fraught with challenges. The effectiveness of CO_2_ photoreduction is limited by the challenging chemisorption and activation of CO_2_ molecules on the catalyst, mainly due to the high dissociation energy of C = O bond.^[^ ^4^ ^]^ Furthermore, current photocatalysts suffer from critical limitations such as inefficient solar energy utilization, excessive recombination of photogenerated electron‐hole pairs, and substantial internal resistance to charge carrier migration.^[^ ^5^ ^]^ Photocatalysts serve as pivotal mediators in CO_2_RR, governing the efficiency and selectivity of the reaction through their intrinsic electronic structures, surface reactivity, and light‐harvesting capabilities.^[^ ^6^ ^]^ Therefore, identifying suitable photocatalysts is a pressing issues that require immediate attention. Among them, layered double hydroxides (LDHs) have exhibited promising performance in the field of photocatalysis, thereby attracting extensive research attention.

LDHs are nanostructured 2D layered solids with the general formula [M^2+^_1‐x_M^3+^_x_(OH)_2_]^y+^(A^z−^)·nH_2_O, where M^2+^ typically represents Ca^2+^, Mg^2+^, Ni^2+^, Zn^2+^, or Co^2+^; M^3+^ denotes Al^3+^ or Fe^3+^; y = x; and A is a charge‐balancing anion, commonly carbonate.^[^ ^7^, ^8^ ^]^ LDHs demonstrate significant advantages in photocatalytic applications, primarily stemming from their atomic thickness and excellent mechanical, electrical, and thermal properties combine to achieve new functions.^[^ ^9^, ^10^, ^11^ ^]^ LDHs are recognized as highly efficient 2D photocatalysts^[^ ^12^ ^]^ that exhibit high light absorption efficiency. The nanosheet structure of LDHs shortens the migration distance of photo‐generated carriers,^[^ ^13^ ^]^ yet their narrow band gaps contribute to elevated electron‐hole recombination rates. Due to they are ease to design and adjustment,^[^ ^14^ ^]^ constructing heterojunctions by integrating LDHs with other semiconductor materials with a wide bandgap facilitates photogenerated carrier separation and mitigates electron‐hole recombination, while the inherent basicity of LDHs enhances CO_2_ adsorption on the composite photocatalyst surface.^[^ ^15^ ^]^ For instance, Liu et al.^[^ ^16^ ^]^ prepared a composite catalyst CoAl‐LDH@CdS, which enhanced charge transfer and photodegradation activity. The composite catalyst conforms to the Type‐II heterostructure, which reduces the recombination rate of photo‐generated electrons. However, the oxidation‐reduction reaction occurs on the semiconductor catalyst at a lower potential, and the reduction efficiency needs to be improved.^[^ ^17^ ^]^ Santosh Kumar et al.^[^ ^15^ ^]^ has designed a p‐n heterojunction catalyst P25@CoAl‐LDH, whose nanostructure provides abundant active sites for the reduction reaction, and has excellent activity and CO selectivity of more than 90% in the CO_2_RR process without the use of sacrificial agents. The optimal composite material exhibits a CO generation rate that is 2.5‐fold and 5.5‐fold enhanced relative to single CoAl‐LDH and P25, respectively. This composite heterojunction is also consistent with a type II heterojunction mechanism. Furthermore, Huiyin Ye et al.^[^ ^18^ ^]^ constructed a 2D/2D S‐scheme Bi_2_MoO_6_/CoAl‐LDH heterojunction, where the optimized BMO/CoAl_30_ sample demonstrated a TC photodegradation rate constant of 3.637×10^−2^ min^−1^, representing 4.01 times and 1.26 times enhancements over pristine CoAl‐LDH and Bi_2_MoO_6_, respectively. This S‐scheme heterostructure maintained high light absorption efficiency while preserving strong redox potential, thereby significantly boosting catalytic performance.

Semiconductor nanostructures with wide band gaps are widely used in photocatalysis. Among these, Zirconium dioxide (ZrO_2_) is a non‐toxic, low‐cost, and good thermal stability n‐type semiconductor.^[^ ^19^ ^]^ Notably, Herrmann et al.^[^ ^20^ ^]^ reported ZrO_2_’s photocatalytic activity for hydrogen evolution via water splitting under UV irradiation. However, the limited solar utilization efficiency resulting from the UV portion constituting only 4%–5% of the solar spectrum leads to suboptimal photocatalytic performance. It is concluded that although ZrO_2_ exhibits robust redox capability and effective suppression of electron‐hole recombination, its inherent wide bandgap results in limited light absorption capacity, creating a performance trade‐off that impacts photocatalytic reduction efficiency.^[^ ^21^, ^22^ ^]^ Notably, when combined with other active ingredients, it can act as a catalyst or a catalytic substrate to effectively improve photocatalytic performance through the construction of heterojunctions. For instance, a Z‐scheme ZrO_2_‐TaON photocatalytic system^[^ ^23^ ^]^ markedly boosts the hydrogen evolution efficiency of TaON under visible light irradiation. Zhang et al.^[^ ^24^ ^]^ prepared an S‐scheme g‐C_3_N_4_/ZrO_2_ composite with rapid electron migration and increased effective utilization of photogenerated electrons under the strong internal electric field (IEF) of S‐scheme heterojunctions compared with pristine ZrO_2_. ZrO_2_ has been reported many times in the field of photocatalytic hydrogen production, but it is rarely applied in the field of photocatalytic reduction of CO_2_ to produce CO. Due to the high positive valence band (VB) of ZrO_2_, it has strong oxidation capacity and excellent stability. Its low conduction band (CB) restricts the reduction of electrons, but makes the crucial S‐scheme recombination possible.

Building on these findings, this study employs a hydrothermal method to grow CoAl‐LDH nanosheets on bulk ZrO_2_. The nanosheet architecture and heterojunction interface synergistically expand interfacial contact between semiconductors and enhance the catalyst's specific surface area, which in turn facilitates the creation of a high density of active sites for CO_2_RR. This S‐scheme heterojunction establishes a robust internal electric field (IEF) that accelerates charge transfer kinetics, leading to further enhancement of CO_2_RR efficiency. Especially, the optimal LZ‐60 composite catalyst achieves a remarkable CO evolution rate of 562.545 mmol^−1^ g^−1^ h^−1^, representing 5 times and 43 times improvements over pristine CoAl‐LDH and ZrO_2_, respectively. X‐ray photoelectron spectroscopy (XPS) tests and density functional theory (DFT) calculations provide effective verification of the electron transfer process in the S‐scheme heterojunction. In situ Fourier transform infrared (FTIR) characterization elucidates the CO production pathway in the CO_2_RR process.

## 2 Experimental Section

### 2.1 Main Experimental Materials

All materials and sources of chemicals are shown in Text S1 (Supporting Information). The reagents listed above can be directly employed in the reaction without additional purification steps.

### 2.2 CoAl‐LDH Synthesis

CoAl‐LDH was synthesized via a hydrothermal method. A mixture of 1.5 mmol Co(NO_3_)_2_·6H_2_O, 0.5 mmol Al(NO_3_)_3_·9H_2_O, 2 mmol NH_4_F, and 5 mmol urea was stirred magnetically, followed by hydrothermal treatment at 100 °C for 24 h. After cooling to room temperature, the product was washed several times with deionized water and then centrifuged. The obtained pink powder was then dried in a vacuum oven at 80 °C for 12 h to get CoAl‐LDH.

### 2.3 ZrO_2_ Synthesis

ZrO_2_ nanoparticles were prepared using the facile precipitation method. First, 3.2 g of ZrOCl_2_·8H_2_O was added to 50 mL of ultrapure water and stirred magnetically for 15 min. Then, a 1 m NaOH solution was added dropwise and stirred until the pH of the solution was adjusted to 11–12. The white deposits that separated from the water were washed once or twice with ethanol and ultrapure water until no metal salts remained. The product was then dried in an oven at 80 °C for 12 h, annealed in a muffle furnace at 350 °C for 4 h, and subsequently dried at 80 °C for 6 h.

### 2.4 CoAl‐LDH/ ZrO_2_‐*x* (LZ‐*x*) Synthesis

**Figure 1:**
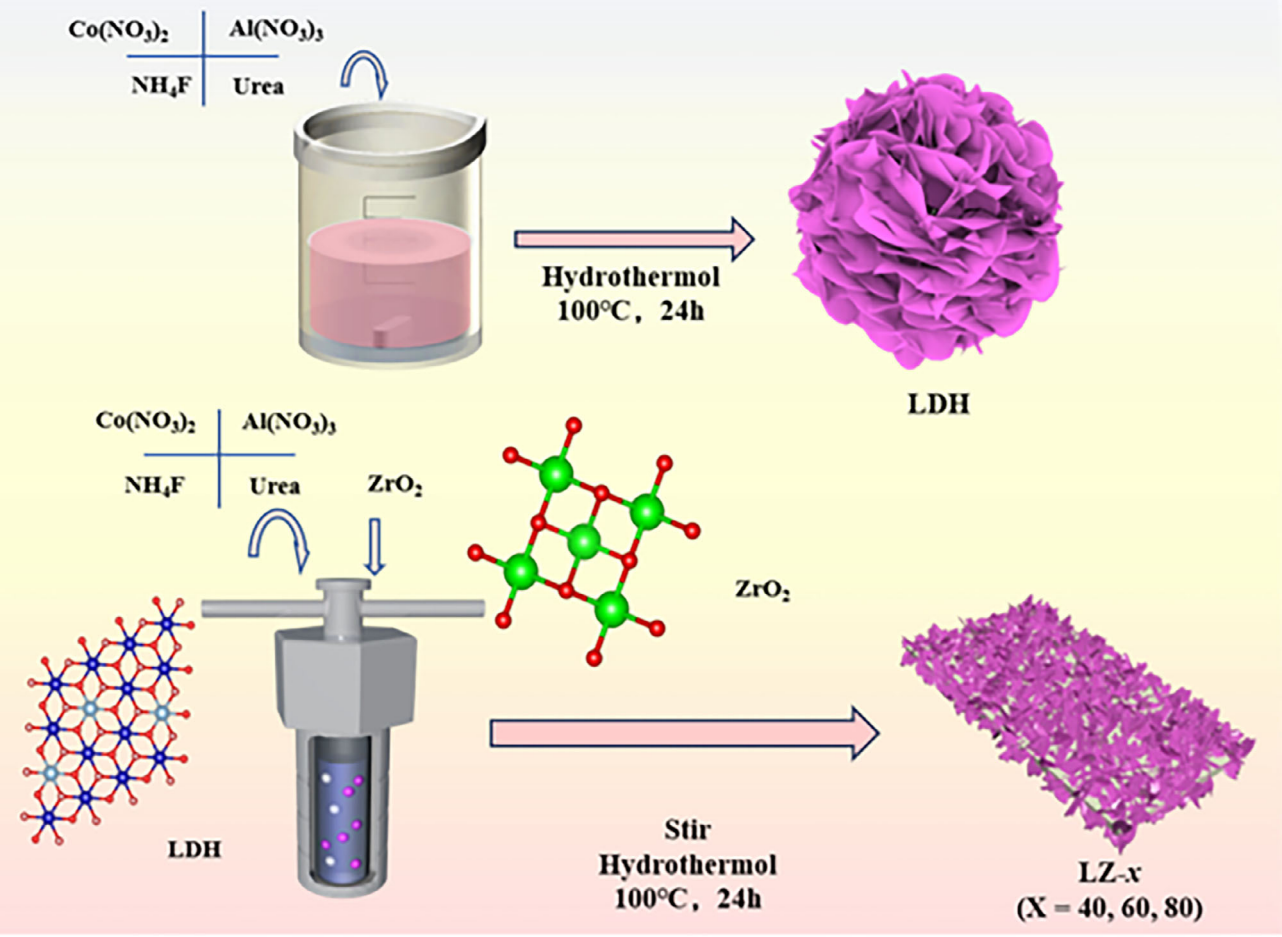
Preparation process of CoAl‐LDH and a series of LZ‐ *x*.

As shown in **Figure** **1** CoAl‐LDH/ZrO_2_ composites (LZ‐*x*, *x*  =  40, 60, 80 mg ZrO_2_) were prepared by in situ growth of CoAl‐LDH nanosheets on ZrO_2_ substrates via hydrothermal synthesis. A precursor solution containing Co(NO_3_)_2_·6H_2_O, Al(NO_3_)_3_·9H_2_O, NH_4_F, and urea was stirred until homogeneous. The synthesized ZrO_2_ powder (40/60/80 mg) was added, then the mixed solution was transferred into Teflon‐lined stainless‐steel autoclaves and subjected to hydrothermal treatment at 100 °C for 24 h. The products were washed, centrifuged, and dried at 80 °C under vacuum; the obtained samples were labeled as LZ‐40, LZ‐60, or LZ‐80 based on ZrO_2_ content.

### 2.5 Catalyst Characterization

Detailed methods are provided in Text S2 (Supporting Information).

### 2.6 Computational Method and Models

Detailed methods of DFT calculation are provided in Text S3 (Supporting Information).

### 2.7 Catalytic Performance Test

Detailed methods are provided in Text S4 (Supporting Information).

## 3 Results and Discussion

### 3.1 Catalyst Morphology

**Figure 2:**
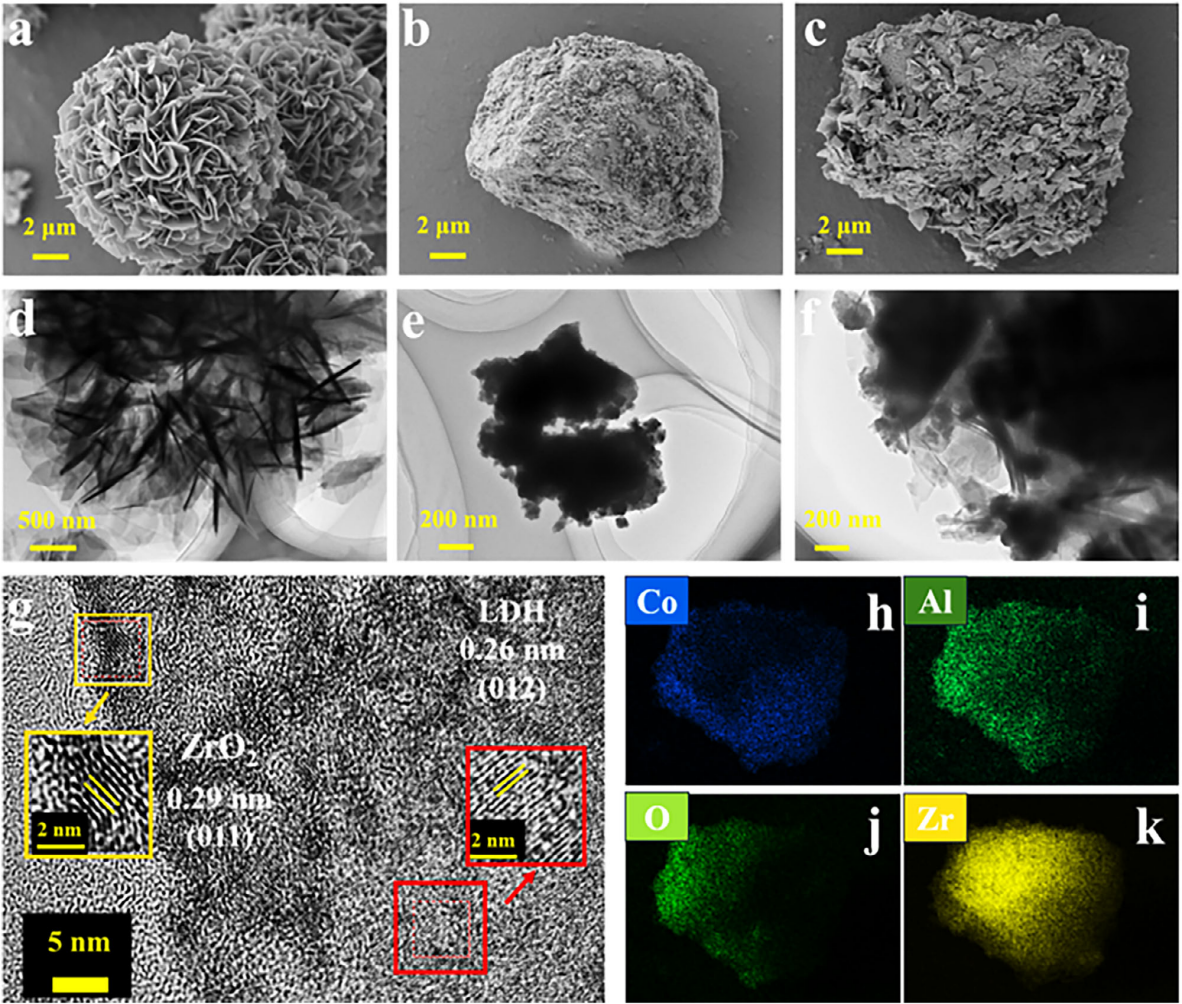
SEM images of a) CoAl‐LDH, b) ZrO_2_, and c) LZ‐60. TEM images of d) CoAl‐LDH, e) ZrO_2_, f) LZ‐60. g) HRTEM images of LZ‐60. h–k) Elemental mapping images of LZ‐60.

The morphology of CoAl‐LDH, ZrO_2_, and their composites was characterized using scanning electron microscopy (SEM). Pure CoAl‐LDH consists of uniformly assembled nanosheets (100–200 nm) with an overall flower morphology (**Figure** **2**a). ZrO_2_ displayed a stacked aggregate structure of irregular nanoparticles (Figure 2b). Following the growth of CoAl‐LDH on the ZrO_2_ substrate, the lamellar nanosheets of CoAl‐LDH are inserted on the irregularly stacked bulk ZrO_2_. The addition of ZrO_2_ affects the growth of CoAl‐LDH, and the flower‐like microsphere structure is dispersed into the nanosheets that are tightly bonded to the ZrO_2_ and dispersed on the surface of the ZrO_2_ homogeneously, to form a strong adhesion aggregation (Figure 2c). The tight junction at the heterojunction interface is a favorable factor for accelerated electron transfer and promotes the separation of photogenerated carriers. ZrO_2_ provides an ideal loading platform for the growth of CoAl‐LDH nanosheets, effectively preventing their agglomeration. The SEM of LZ‐40 and LZ‐80 is shown in Figure S1 (Supporting Information). All the structures presented by CoAl‐LDH/ZrO_2_ are conducive to increasing interfacial contact, enriching reactive sites, and facilitating the separation of photogenerated charges.

Transmission electron microscopy (TEM) revealed the nanoscale structural features of CoAl‐LDH, ZrO_2_, and the LZ‐60 composite (Figure 2d–f). Pristine CoAl‐LDH exhibited detached nanosheets (100–200 nm) derived from the parent microspheres (Figure 2d), while ZrO_2_ exhibits a block stacking (Figure 2e). The LZ‐60 heterostructure showed CoAl‐LDH nanosheets intercalated within stacked bulk ZrO_2_ regions, with contrast differentiation indicating ZrO_2_ (dark regions) and CoAl‐LDH (gray regions) (Figure 2f). High‐resolution TEM (HRTEM) was employed to visualize the interfacial structure of the composite samples. HRTEM of LZ‐60 (Figure 2g) confirmed intimate interfacial contact, with measured lattice fringes of 0.26 and 0.29 nm corresponding to the CoAl‐LDH (012) and ZrO_2_ (011) planes, respectively. This structural coherence validates successful heterojunction formation and optimal carrier transfer pathways. Additionally, the Energy‐dispersive X‐ray spectroscopy (EDS) mapping (Figure 2h–k) confirmed the coexistence of Co (blue), Al (green), O (bright green), and Zr (yellow) across the LZ‐60 composite surface. Quantitative elemental composition (Table S1, Supporting Information) further corroborated the successful integration of CoAl‐LDH and ZrO_2_ phases, validating heterostructure formation.

**Figure 3:**
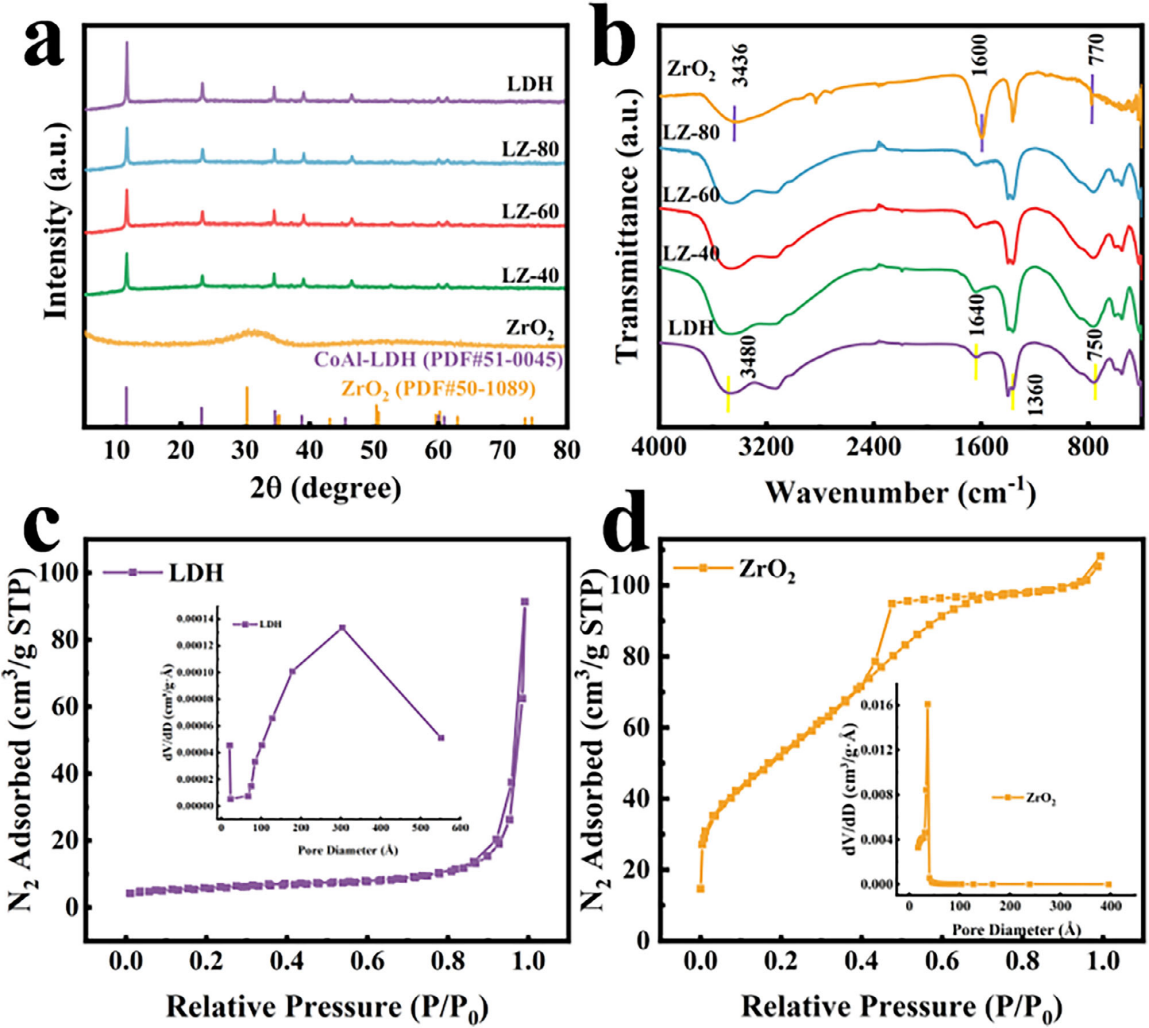
a) XRD patterns of CoAl‐LDH, LZ‐*x*, and ZrO_2_. b) FTIR spectra. N_2_ adsorption‐desorption isotherms of c) CoAl‐LDH, d) ZrO_2_.

X‐ray diffraction (XRD) patterns (**Figure** **3**a) confirm the phase composition of the synthesized materials. Pristine CoAl‐LDH exhibits characteristic peaks at 11.5°, 23.2°, 34.6°, and 38.7°, indexed to the (003), (006), (012), and (015) planes of hexagonal Co_6_Al_2_CO_3_(OH)_16_·4H_2_O (PDF#51‐0045),^[^ ^25^ ^]^ validating its successful synthesis. The LZ‐40, LZ‐60, and LZ‐80 composites show XRD patterns nearly identical to pristine CoAl‐LDH, with no new peaks detected, indicating preserved CoAl‐LDH structure upon ZrO_2_ integration. Bare ZrO_2_ displays a broad diffraction peak at 32° (011), consistent with poorly crystalline ZrO_2_ (PDF#50‐1089).^[^ ^26^ ^]^ XRD characterization of LZ‐*x* heterostructures reveals preserved CoAl‐LDH crystallinity postcomposite formation, with unaltered diffraction peaks at 11.5°, 23.2°, 34.6°, and 38.7°. However, peak intensities decrease progressively with increasing ZrO_2_ content, likely due to reduced interlayer spacing from ZrO_2_ incorporation. Notably, no distinct ZrO_2_ diffraction peaks are observed in LZ‐*x* composites, attributed to either low ZrO_2_ loading or its poor crystallinity. This absence is corroborated by prior SEM and TEM imaging confirming ZrO_2_ presence. All samples exhibit phase‐pure structures matching standard references, indicating high sample purity.

Fourier transform infrared (FTIR) was performed on all samples to investigate the chemical bonds and functional groups of the catalysts. As shown in Figure 3b, the peaks located at ≈3480 and 1640 cm^−1^ are caused by the stretching vibrations of O‐H bonds within the host layers and interlayers of CoAl‐LDH, as well as the bending vibrations of H_2_O.^[^ ^27^ ^]^ The peak observed at ≈1360 cm^−1^ corresponds to the stretching ν_3_ antisymmetric stretch of the carbonate ion (${\mathrm{CO}}_{3}^{2-}$) attributed to the CoAl‐LDH structure. Additionally, the band near 750 cm^−1^ is assigned to the ν_2_ out‐of‐plane bend of M‐OH and M‐O vibrations mode. (where M represents metal elements, specifically Co and Al in this context).^[^ ^28^ ^]^ FTIR analysis of ZrO_2_ nanoparticles reveals characteristic absorption bands, the peaks at 3436 and 1600 cm^−1^ correspond to O‐H stretching vibrations from adsorbed H_2_O on the ZrO_2_ surface, while the 527 cm^−1^ band arises from Zr‐O bond stretching vibrations. The absorption features at 770 and 457 cm^−1^ are attributed to Zr‐O‐Zr bridge stretching modes.^[^ ^29^ ^]^ Upon doping with ZrO_2_, the CoAl‐LDH/ZrO_2_ composites exhibit a slight red shift of these functional group peaks, suggesting potential heterojunction interactions. Notably, the FTIR spectrum of the CoAl‐LDH/ZrO_2_ composite retains the original CoAl‐LDH framework while manifesting weak Zr‐O stretching modes, confirming the formation of a CoAl‐LDH/ZrO_2_ heterostructure with preserved component characteristics and emergent interfacial interactions.

Nitrogen adsorption‐desorption isotherms measured at 77 K (Figure 3c,d) revealed Type IV isotherms with H3‐type hysteresis loops (IUPAC classification)^[^ ^30^ ^]^ for pristine CoAl‐LDH, ZrO_2_, and LZ composites, indicative of mesoporous structures with slit‐like pores. The Brunauer‐Emmett‐Teller (BET) diagrams of LZ‐40, LZ‐60, and LZ‐80 are shown in Figure S2 (Supporting Information). Capillary condensation occurs on the adsorption isotherm within the range of 0.45–1.0 relative pressure of nitrogen P/P_0_. This phenomenon enables the condensation of N_2_ molecules at atmospheric pressure, thereby filling the hysteresis loops formed by the mesoporous channels. This observation suggests that the composites possess a substantial number of mesopores with irregular pore structures, characterized by flat seams, cracks, crazes, or wedges in the form of sliced layers.^[^ ^31^ ^]^ The pore size distribution of all samples is predominantly within the range of 2–40 nm. As shown in Table S2 (Supporting Information), the specific surface areas are ranked according to the BET method: ZrO_2_ > LZ‐80 > LZ‐60 > LZ‐40 > CoAl‐LDH. The specific surface area of the photocatalyst grows with increasing ZrO_2_ content from 19.8915 m^2^ g^−1^ (CoAl‐LDH) to 68.9505 m^2^ g^−1^ (LZ‐80), indicating that introducing ZrO_2_ into composites with abundant mesopores effectively increases the specific surface area compared to CoAl‐LDH. The composite contributes to the increase in the surface area of the heterojunction catalyst and builds up the number of active sites for photocatalytic reactions, which makes a certain contribution to the catalytic performance. Additionally, the average pore size of ZrO_2_, CoAl‐LDH, LZ‐40, LZ‐60, and LZ‐80 was 3.3006, 36.5472, 33.7527, 8.8414, and 7.1291 nm, respectively. The measured pore volumes were 0.1674, 0.1412, 0.1734, 0.1126, and 0.1169 cm^3^ g^−1^ for the respective catalysts. The pore volume of CoAl‐LDH/ZrO_2_ decreased due to an increase in main micropores, as confirmed by the pore volume distribution curve. Compared with CoAl‐LDH, CoAl‐LDH/ZrO_2_ has a higher specific surface area and more suitable mesoporous structure characteristics. Notably, the photocatalytic activity of ZrO_2_ was extremely low despite its largest surface area of 179.7079 m^2^ g^−1^, suggesting that photocatalytic activity is associated with electron migration and electron‐hole pair complexation. Together with the BET test and reduction performance results, it was determined that the specific surface area and pore volume of the photocatalysts were not critical for photocatalytic activity.

### 3.2 Surface Chemical State

We characterized the chemical states of the elements on the sample surface using X‐ray photoelectron spectroscopy (XPS). The survey spectrum (Figure S3a, Supporting Information) shows the results for all elements detected in the two original catalysts and the composite catalyst. LZ‐60 contains the elements Co, Al, Zr, and O, which is consistent with the EDS results and prove that the composite catalysts were successfully prepared.

All XPS spectra are referenced to amorphous carbon (C 1s 284.8 eV). The XPS spectra of C1s in CoAl‐LDH, LZ‐60, and ZrO_2_ are presented in Figure S3b (Supporting Information). The peaks at binding energies of 284.82, 284.83, and 285.07 in CoAl‐LDH, LZ‐60, and ZrO_2_ are commonly regarded as C‐C bonds.^[^ ^25^ ^]^ The peaks at 288.48 and 288.50 for CoAl‐LDH and LZ‐60 are due to the carbonate^[^ ^32^ ^]^ present between the layers of CoAl‐LDH. The peak at 282.42 eV for LZ‐60 likely indicates the formation of C‐Zr bonds. Meanwhile,^[^ ^33^ ^]^ the peak at 287.28 eV for ZrO_2_ signifies C‐O bonds.^[^ ^34^ ^]^

**Figure 4:**
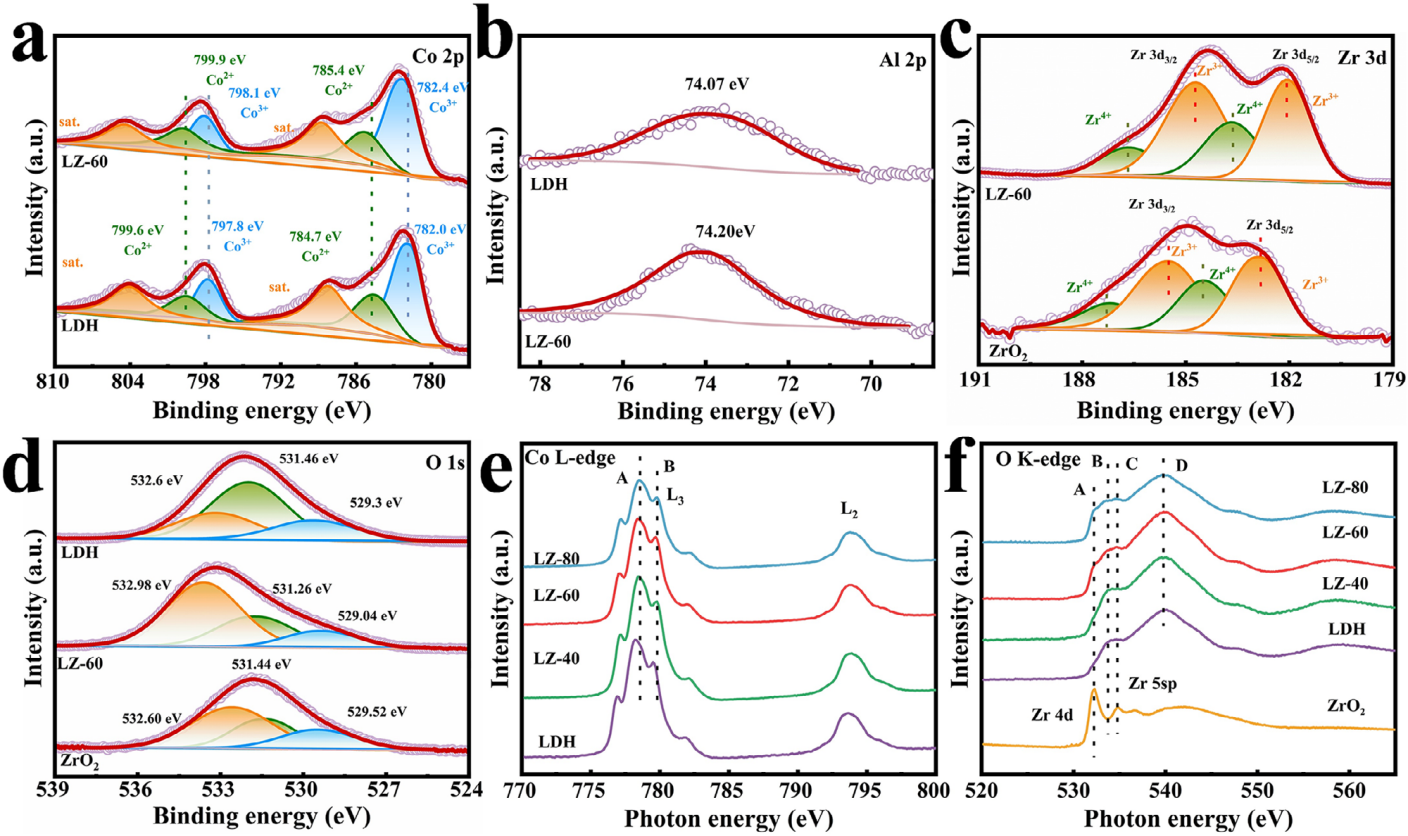
XPS spectra of a) Co 2p, b) Al 2P, c) Zr 3d, d) O 1s. sXAS of e) Co L‐edge, f) O K‐edge.

Co elements corresponding to CoAl‐LDH and LZ‐60 in **Figure** **4**a. The peaks at 782.0 and 797.8 eV are considered to represent the Co^3+^ 2p_3/2_ and 2p_1/2_ orbitals of CoAl‐LDH, and the peaks at 784.7 and 799.6 eV indicate the Co^2+^ 2p_3/2_ and 2p_1/2_ orbitals. The 782.4 and 798.1 eV peaks in LZ‐60 are attributed to Co^3+^ 2p_3/2_ and 2p_1/2_, respectively; the 785.4 and 799.9 eV peaks are thought to be Co^2+^ 2p_3/2_ and 2p_1/2_, with the binding energy of elemental cobalt increased compared to CoAl‐LDH. The peaks located at 788.3 and 804.1 eV were labeled as Co^2+^ and Co^3+^ for CoAl‐LDH. The two peaks at 788.8 and 804.5 eV corresponded to Co^2+^ and Co^3+^ for LZ‐60.

As shown in Figure 4b, the Al element has a 2p peak at 74.07 eV in CoAl‐LDH, which corresponds to Al^3+^, and after the addition of ZrO_2_ the 2p peak of Al is altered and shifted to 74.20 eV toward higher binding energy. The elevated binding energy of the composite catalyst Al indicates that CoAl‐LDH loses electrons during the construction of the heterojunction with ZrO_2_.

The Zr 3d signal can be deconvoluted into four bands at 186.7 and 184.7 eV (3d_3/2_), as well as 183.7 and 182.1 eV (3d_5/2_) (Figure 4c). These correspond to Zr^3+^ and Zr^4+^. The relatively large proportion of Zr^3+^ in LZ‐60 indicates the presence of abundant oxygen vacancies.^[^ ^35^ ^]^ Some oxygen atoms were removed from the synthesized LZ‐60 material, significantly changing the local electron distribution. The abundant oxygen vacancies contribute to the adsorption and activation of CO_2_ and H_2_O. Upon complexation with CoAl‐LDH, both Zr 3d_3/2_ and Zr 3d_5/2_ peaks were slightly shifted to lower binding energies, suggesting that electrons may be transferred from CoAl‐LDH to ZrO_2_ sites.^[^ ^36^ ^]^ This result also implies the existence of LZ‐60 interaction with oxygen‐rich vacancies.

O element analysis by XPS was performed on each sample (Figure 4d). The O 1s orbitals of the CoAl‐LDH were categorized as 529.30, 531.46, and 532.60 eV. These were attributed to unsaturated oxygen ligands,^[^ ^37^ ^]^ the OH^−^ in the LDH, and defective sites of adsorbed oxygen,^[^ ^38^ ^]^ respectively. The O 1s orbital situation of LZ‐60 was almost the same as that of the CoAl‐LDH; however, the peak area of the defective sites of the adsorbed oxygen increased. These sites are favorable for CO_2_ adsorption and reaction activation. For ZrO_2_, the binding energies of 529.52, 531.44, and 532.60 eV are related to lattice oxygen at O_v_, surface‐adsorbed oxygen at O_v_, and hydroxyl oxygen,^[^ ^39^ ^]^ respectively. The EPR characterization of ZrO_2_ is provided in Figure S4 (Supporting Information). The signals of ZrO_2_ at g = 2.003 could be identified as the electrons trapped on oxygen vacancies, indicating that ZrO_2_ has oxygen vacancies.

From this, it can be determined that the Co and Al elements in CoAl‐LDH move toward higher binding energy after composite with ZrO_2_, while the Zr element moves toward lower binding energy after composite, which indicates that there is a strong interaction between CoAl‐LDH and ZrO_2_, and the e^−^ is transferred from CoAl‐LDH to ZrO_2_, which establishes a strong IEF inside the composite catalyst. These once again proved that the constructed S‐scheme heterojunction was established.

Soft X‐ray absorption spectroscopy (sXAS) is an effective means for detecting surface valence states and surface chemical environments with high sensitivity. The oxidation states of the surface elements of the prepared samples were further probed by sXAS characterization, and the transition metal L_2,3_‐edge XAS spectra were sensitive to changes in oxidation states, spin states, or orbitals. The L‐edge XAS of Co was divided into L_2_ and L_3_ regions, corresponding to the leaps from the 2p_1/2_ and 2p_3/2_ energy levels to unoccupied 3d orbitals, respectively.

As illustrated in Figure 4e, the L_2_ and L_3_ edge sXAS spectra of elemental Co are presented. In the case of 3d transition metals, the L‐side sXAS represents the electron transfer from 2p to the unoccupied 3d orbitals. The single CoAl‐LDH and each composite sample demonstrate the characteristic peaks of the mixed state of Co^2+^ and Co^3+^.^[^ ^40^ ^]^ It is noteworthy that the intensity of Co^2+^ (peak A) decreases relative to that of Co^3+^ (peak B) when CoAl‐LDH is complexed with ZrO_2_, suggesting that charge transfer occurs with the establishment of the heterojunction. During the photocatalytic reduction of CO_2_, the positively charged Co may act to lower the charge transfer potential barrier and thus increase the photocatalytic activity. The combination of CoAl‐LDH with ZrO_2_ results in a decrease in the intensity of the characteristic peaks of the L‐edge sXAS pattern of Co. In addition, the peak position of the composite catalyst is shifted toward higher energies compared to the original catalyst. The presence of more occupied 3d orbitals at higher energies suggests that the valence state of Co is elevated after complexation. Therefore, it can be concluded that charge redistribution occurred during the hybridization process, corresponding to the conclusion obtained from XPS characterization.

As shown in Figure 4f, the K‐edge sXAS results of the oxygen (O) elements reveal differences in their chemical coordination environments in CoAl‐LDH and ZrO_2_. ZrO_2_ exhibits a stronger peak at ≈533 eV, while CoAl‐LDH shows a broader peak. The A peak in ZrO_2_ at 533 eV is believed to be related to the hybridization of the Zr 4d and O 2p orbitals. The B peak in the spectrogram is believed to be the hybridization orbital of the Co 3d and O 2p orbitals in CoAl‐LDH and LZ‐*x*. The C peak is usually a hybridization orbital peak between the 3p orbitals of elemental Al and the 2p orbitals of elemental O, as well as surface adsorption of oxygen species. The D peaks in CoAl‐LDH and LZ‐*x* are attributed to covalent peaks of Co 4sp/Zr 5sp hybridized orbitals and O 2p orbitals.^[^ ^41^ ^]^ As the proportion of ZrO_2_ in the composite catalysts increases, the A peak's intensity gradually increases while its position shifts slightly toward higher energies. Thus, charge transfer from CoAl‐LDH to ZrO_2_ injects vitality into the lattice oxygen of both materials.^[^ ^42^ ^]^

**Figure 5:**
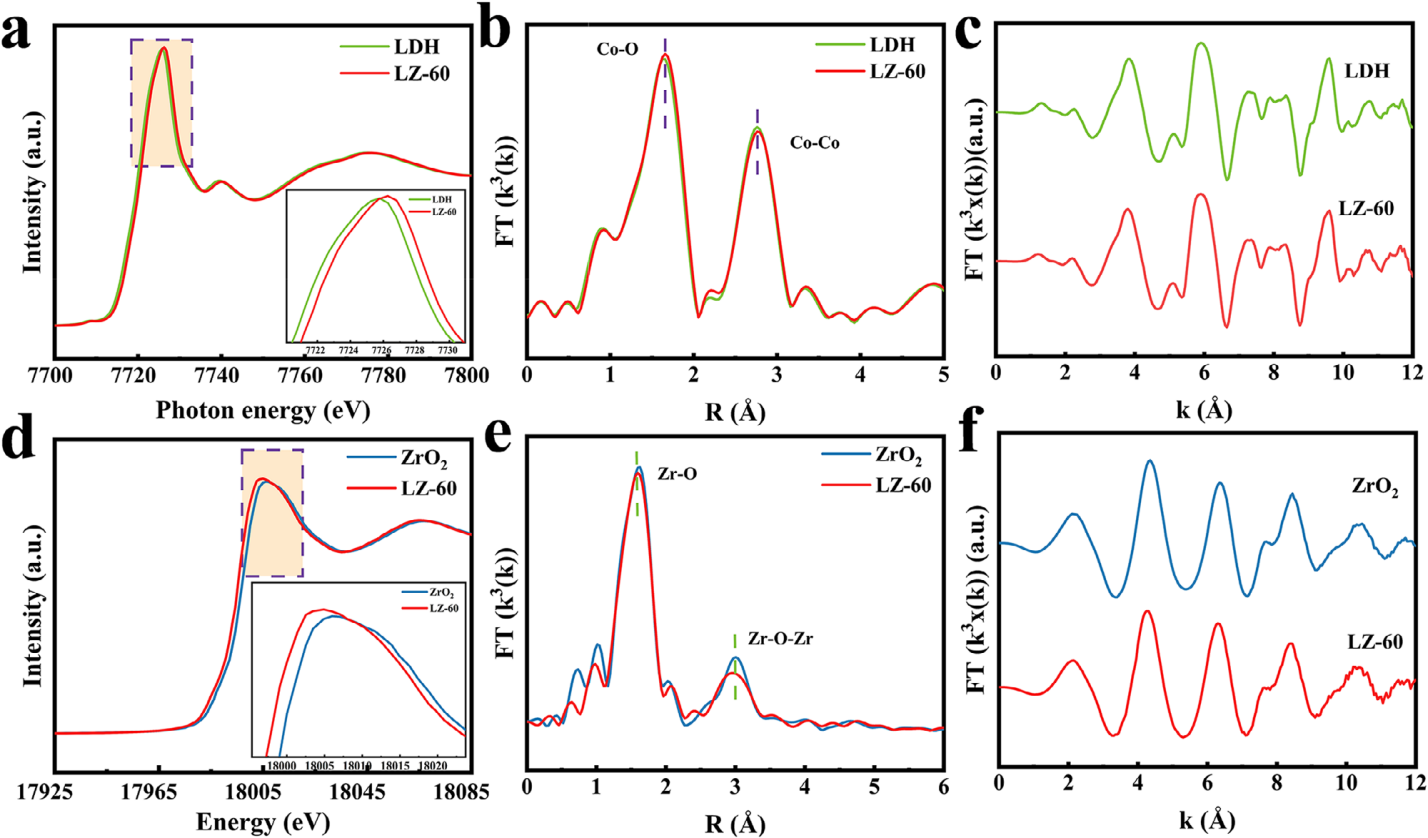
XANES spectra of a) Co K‐edge and d) Zr K‐edge. EXAFS spectra over R‐space functions of b) Co K‐edge and e) Zr K‐edge. EXAFS oscillation function k^3^χ(k) of c) Co K‐edge and f) Zr K‐edge.

X‐ray absorption fine structure (XAFS) provides detailed information on valence. In conjunction with X‐ray Absorption Near Edge Structure (XANES), the oxidation state of the active element can be determined. The K‐space oscillations of the Co and Zr elements show high signal‐to‐noise ratios (**Figure** **5**c,f). In the context of transition metal elements, a rightward shift in the near‐side absorption edge is indicative of an elevated oxidation state. As demonstrated in Figure 5a, a range of samples demonstrate homologous absorption edge positions in XANES. The increased intensity of the white line peak indicates a shift from the 1s orbital to the unoccupied Co 4p state,^[^ ^43^ ^]^ suggesting that more empty orbitals may improve electron acceptance.

We used extended X‐ray absorption fine structure (EXAFS) spectroscopy to confirm whether the local structure and coordination environment of CoAl‐LDH are similar to those of the composite catalyst LZ‐60. This indicates that the composite catalyst is structurally similar to the pristine catalyst and lays the foundation for further analysis. For the R‐space function of the Co element, the bond lengths of Co‐O at 1.62 Å and Co‐Co at ≈2.74 Å rise slightly, and their strengths were higher than the pristine values. These changes are associated with increased disorder and geometrical distortion,^[^ ^41^ ^]^ suggesting that the supersaturated coordination state consolidates the charge transfer channel for the CO_2_ reduction process (Figure 5b).

Figure 5d shows the Zr K‐edge XANES spectra; the Zr K‐edge of LZ‐60 is close to the Zr K‐edge of ZrO_2,_ indicating that the Zr atoms in LZ‐60 are in an oxidized state. Notably, the slight shift of the Zr‐Kedge to the left indicates that the Zr atoms shift from a high oxidation state (tetravalent) to a low oxidation state (trivalent),^[^ ^44^ ^]^ which is attributed to the heterojunction constituted by the composite with CoAl‐LDH.

We further evaluated the coordination environment around the Zr atom using the Fourier transform (FT) of the k^3^‐weighted EXAFS spectra of the Zr K‐edge (Figure 5e). The Zr‐O coordination in ZrO_2_ corresponds to the shell peak shown at 1.62 Å, while the Zr‐O‐Zr coordination corresponds to the second shell peak shown at 3.02 Å. Meanwhile, the Zr‐O‐Zr bond strength of LZ‐60 is lower than that of ZrO_2_.^[^ ^45^ ^]^ Besides, no peaks corresponding to 2.90 Å were found, indicating that Zr does not exist as metal clusters.

### 3.3 Optical Absorption Properties and Energy Band Structure

**Figure 6:**
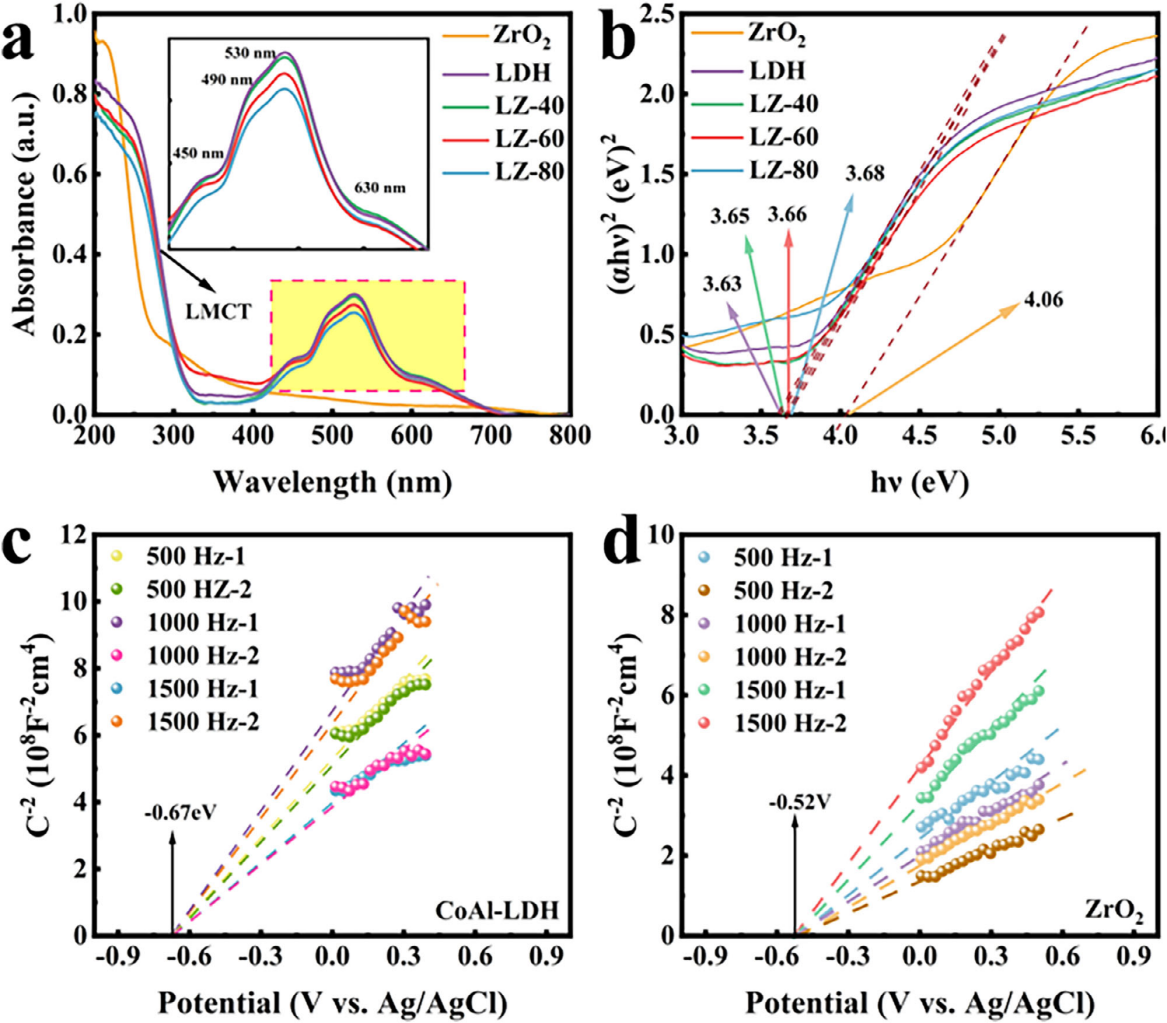
a) UV–vis DRS spectra. b)Tauc spectra. c, d) Mott‐Schottky curves of (c) CoAl‐LDH, (d) ZrO_2_.

The photoabsorption properties of photocatalysts are a key factor in determining their photocatalytic activity. Ultraviolet‐visible diffuse reflectance spectroscopy (UV–vis DRS) was used to determine the light absorption properties of pristine photocatalysts and photocatalysts with different ratios. As shown in **Figure** **6**a, ZrO_2_ exhibits a clear absorption band at 300 nm with no absorption in the visible spectrum. UV–vis DRS was used to investigate the photoresponse properties and band gap information of the catalysts. The pristine CoAl‐LDH exhibited a strong absorption peak in the UV region and a clear absorption edge at 330 nm. The UV region absorption band of CoAl‐LDH is thought to be related to ligand‐metal‐to‐metal charge transfer (LMCT) from O^2−^ to Co^2+^, whereas no metal‐to‐metal charge migration (MMCT) peaks were observed because of the d^0^ electronic configuration of Al^3+^, which usually appear at 264–318 nm.^[^ ^46^ ^]^ The absorption bands in the visible region range from 390 to 780 nm. The d‐d orbital leaps of octahedral Co^2+^ within the CoAl‐LDH layer occur at 450, 490, and 530 nm. The d‐d orbital leaps of octahedrally coordinated Co^3+^ in the low‐spin state show significantly lower absorption peak intensity at 630 nm than Co^2+^, which may be due to its lower content. Heterojunction catalysts exhibit slightly lower absorption intensities in the region from 400 to 600 nm compared to pure CoAl‐LDH. As the CoAl‐LDH content increases, the absorption peak intensity increases and the absorption edge redshifts. Clearly, the introduction of ZrO_2_ optimized the use of sunlight, with a distinct enhancement of absorption from 300 to 400 nm being observed for LZ‐60, corresponding to the best photocatalytic performance.

Tauc plots were obtained from the UV–vis DRS and the Kubelka‐Munk equation. Band gap values were calculated for all samples using Equation (1).

(1) $${\left(\alpha h\nu \right)}^{n}=K\left(h\nu -E_{g}\right)$$

where ν is the frequency of the absorbed light, *h* is Planck's constant, *K* is the scaling factor, *α* is the absorption coefficient, and *E*_g_ is the bandgap energy (eV), with n being 1/2 for indirect bandgap semiconductors and 2 for direct bandgap semiconductors. Graphs are plotted with hν as the horizontal coordinate and (αhν)^n/2^ as the vertical coordinate, and the value of the intersection of the slant tangent line and the horizontal axis is the bandgap energy.

As shown in the Figure 6b, the band gap values of ZrO_2_, LDH, LZ‐40, LZ‐60, and LZ‐80 are 4.06, 3.63, 3.65, 3.66, and 3.68 eV, respectively. In order to understand the electron transfer mechanism as well as the energy band structure within the composite catalysts and to confirm the mechanism of heterojunction compliance, more detailed information is required, and therefore, the flat band potentials of the two semiconductors have been determined by means of Mott‐Schottky (M‐S) diagrams (Figure 6c,d). Six M‐S tests were carried out for CoAl‐LDH and ZrO_2_ under a three‐electrode system at frequencies of 500, 1000, and 1500 Hz, respectively. The slopes of the M‐S plots for CoAl‐LDH and ZrO_2_ were positive, indicating that both materials are n‐type semiconductors. The points where the M‐S curves intersect the transverse axes were obtained. The Flat band potentials (E_fb_) of CoAl‐LDH and ZrO_2_ are −0.67 and −0.52 eV (vs Ag/AgCl), respectively, which are −0.47 and −0.32 eV (vs NHE). Typically, E_fb_ (NHE) is approximately equal to the Fermi energy level (E_f_) value. Therefore, the E_f_ values for CoAl‐LDH and ZrO_2_ are −0.47 and −0.32 eV, respectively. The E_fb_ of n‐type semiconductors is usually considered to be very close to the E_CB_, which is 0.1 eV higher than the E_fb_. Thus, the E_CB_ of CoAl‐LDH is −0.57 eV and the E_g_ is 3.63 eV in the Tauc diagram. The E_VB_ of CoAl‐LDH can be calculated as 3.06 eV by the formula E_VB_ = E_CB_ + E_g_. The E_CB_ of ZrO_2_ is −0.42 eV, and the E_g_ is 4.06 eV. The E_VB_ of ZrO_2_ is 3.64 eV.

Accordingly, energy band structures of composite heterojunction catalysts can be obtained. The band structure of ZrO_2_ and CoAl‐LDH is provided in Figure S5 (Supporting Information). There exists a pronounced alternation in band‐edge potentials between ZrO_2_ and CoAl‐LDH, indicative of a heterojunction interface with modulated carrier dynamics. The imbalance of their chemical potentials leads to the formation of bent energy bands.

### 3.4 Photocatalytic Mechanism

**Figure 7:**
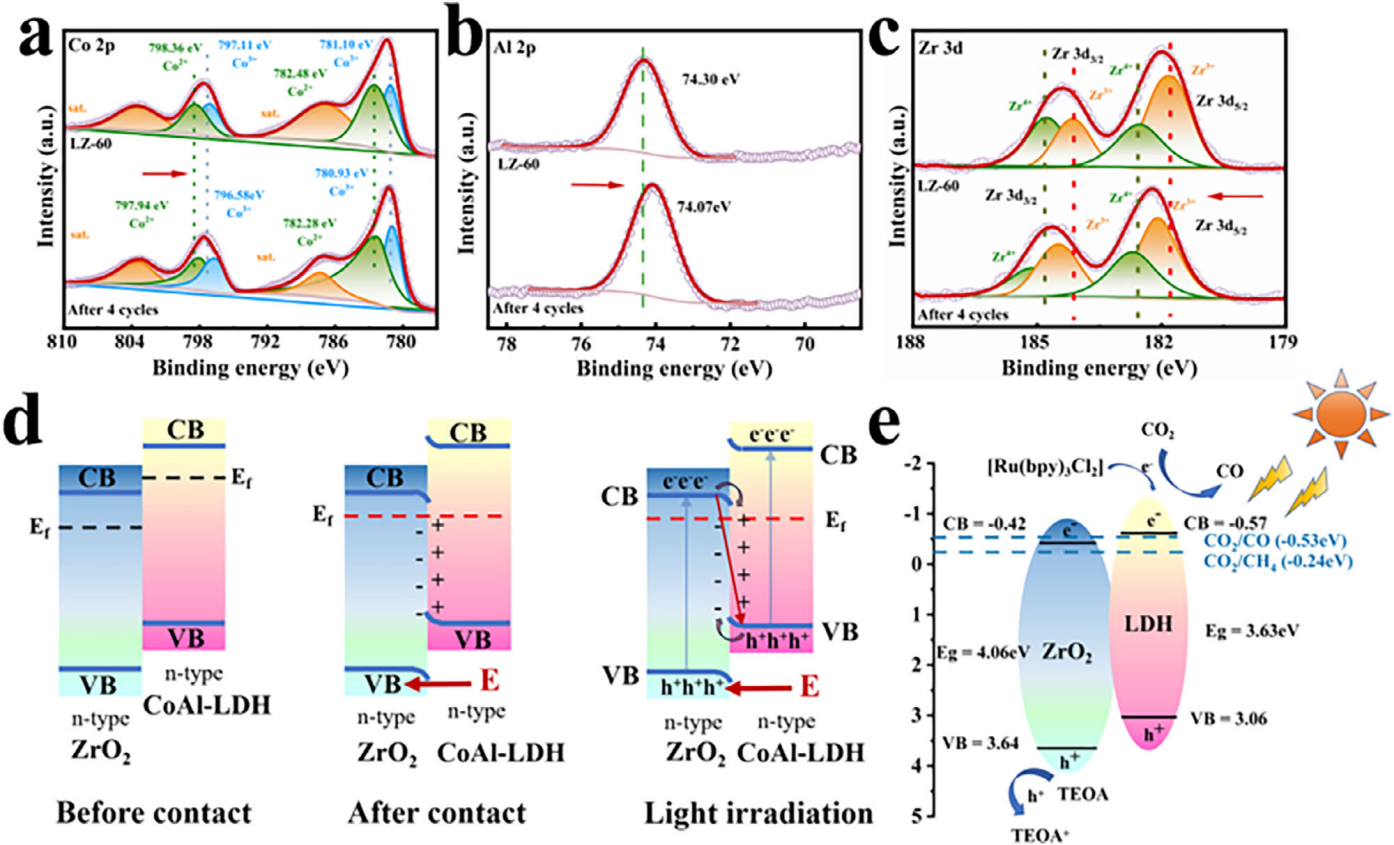
XPS of a) Co, b) Al, c) Zr after CO_2_RR. d) Electronic transfer mechanism of the heterojunction. e) Photocatalytic reduction mechanism of CoAl‐LDH/ZrO_2_.

The constructed composite heterojunction catalysts LZ‐*x* may operate via two distinct mechanisms: a type II heterojunction,^[^ ^47^ ^]^ where the reaction occurs on a semiconductor with a lower redox potential, and an S‐scheme heterojunction, which maximizes photogenerated carrier utilization at higher redox potentials. Photocatalytic reduction involves light‐induced electron migration for CO_2_RR, and XPS spectroscopy is useful to detect this process. After four photocatalytic tests, the Co, Al, and Zr elements of LZ‐60 were analyzed by XPS. As can be seen in **Figure** **7**a, Co 2p_3/2_ reduced from 781.10 to 780.93 eV, and the Co 2p_1/2_ decreased from 797.11 to 796.58 eV. The binding energy of Al decreased from 74.30 to 74.07 eV after several photocatalytic experiments (Figure 7b). Conversely, the binding energy of the Zr element increased, indicating that electrons were transferred from the ZrO_2_ to the CoAl‐LDH under light irradiation (Figure 7c). The binding energy of any element flows in the opposite direction when exposed to light than when it is not exposed to light, suggesting ZrO_2_ can capture holes from the valence band of CoAl‐LDH. These binding energy shifts match the S‐scheme mechanism quite well.^[^ ^48^ ^]^ Furthermore, the E_CB_ of ZrO_2_ is insufficient to reduce CO_2_ to CO. However, a significant amount of CO is present in the previously measured reduction products, indicating that the reduction reaction occurs on the E_CB_ of CoAl‐LDH. Based on analyses of the charge transfer mechanism and band gap calculations along the S‐scheme transfer mechanism, it was found that ZrO_2_ has a lower Fermi energy level (≈−0.32 eV), while CoAl‐LDH has a higher one (≈−0.47 eV). When ZrO_2_ and CoAl‐LDH combine to form a heterojunction, electrons spontaneously transfer from CoAl‐LDH to ZrO_2_ to equalize their Fermi energy levels. During the electron transfer process, an IEF forms from CoAl‐LDH to ZrO_2_, and the energy bands of ZrO_2_ and CoAl‐LDH bend, with the ZrO_2_ energy band bending downward and the CoAl‐LDH energy band bending upward. Figure 7d shows the schematic diagram of the charge transfer of CoAl‐LDH/ZrO_2_ heterojunction. The band bending promotes the recombination of photogenerated electrons in the CB of ZrO_2_ and holes in the VB of CoAl‐LDH in the interface region, which is similar to the principle of water flowing to a lower place.^[^ ^6^ ^]^ The photogenerated electrons in the CB of ZrO_2_ and the holes in the VB of CoAl‐LDH tend to compound at the interface under Coulomb attraction.^[^ ^49^ ^]^ To sum up, the internal electric field, band bending, and Coulomb gravity are the driving forces of CB electron recombination of ZrO_2_ and VB hole recombination of CoAl‐LDH. Therefore, the useless electrons and holes are eliminated by recombination, and the electrons and holes with stronger redox potential are retained to participate in the reaction, so as to improve the photocatalytic reaction ability. When CoAl‐LDH/ZrO_2_ heterojunction catalyst was exposed to the light, it was found that e^−^‐h^+^ pairs were generated on the CB and VB of the composite catalyst (Figure 7e). Due to the IEF force at the interface, CB e^−^ in ZrO_2_ can be easily transferred to the VB of CoAl‐LDH, and compound with holes on VB of CoAl‐LDH, accelerating the separation of e^−^‐h^+^ pairs, and finally leaving electrons on CB of CoAl‐LDH and h^+^ on VB of ZrO_2_, so that CoAl‐LDH can maintain the strong reducibility of CB e^−^, and interact with CO_2_ to produce a large amount of CO, while the oxidation reaction of sacrificial agent is carried out on the VB of ZrO_2_ due to the strong oxidation of h^+^. The above analysis shows that ZrO_2_ and CoAl‐LDH combine to construct a S‐scheme heterojunction photocatalytic reaction system, which has strong redox capability and faster charge carrier separation kinetics, thus greatly improving the photocatalytic reduction efficiency of CO_2_.

### 3.5 Density Functional Theory Computational Calculations

**Figure 8:**
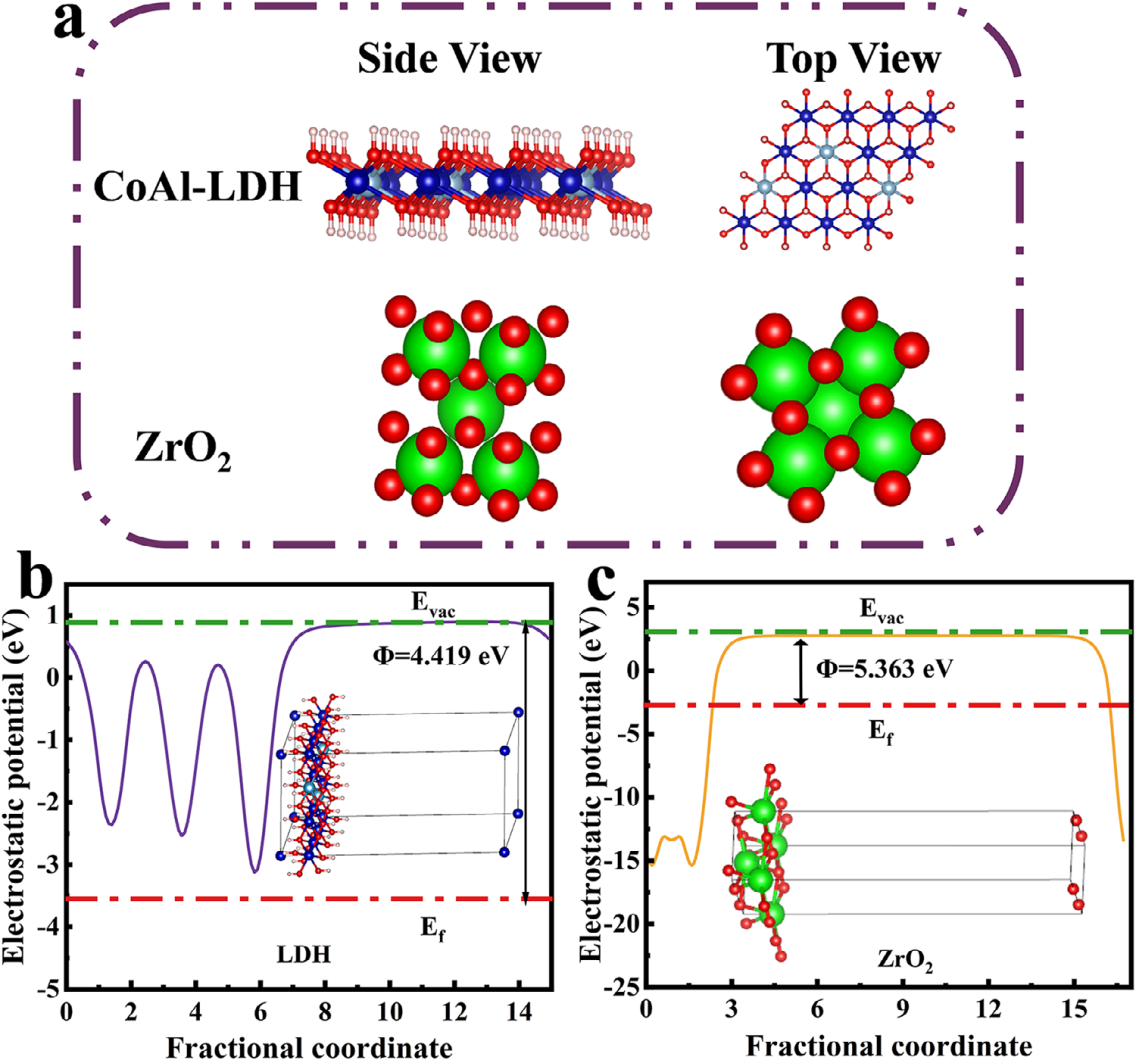
a) Molecular models of CoAl‐LDH and ZrO_2_ for DFT calculations. b,c) The calculated work functions of (b) CoAl‐LDH and (c) ZrO_2_.

DFT calculations were performed to rigorously validate the existence of an IEF at the CoAl‐LDH/ ZrO_2_ interface. The computational models for theoretical analysis are illustrated in **Figure** **8**a, with optimized surface geometries of CoAl‐LDH (012) and ZrO_2_ (011) shown in Figure 8b,c, respectively. The work function (Φ) is the minimum energy required for an electron transfer from the Fermi level (E_f_) to the vacuum level (E_vac_) by photoexcitation. It is calculated as Φ  =  E_vacuum_–E_Fermi_.^[^ ^50^ ^]^ Different materials have different work functions and Fermi energy levels. Prior to combining CoAl‐LDH and ZrO_2_, the work function of CoAl‐LDH (012) was calculated using DFT simulation to be 4.419 eV. This value is significantly smaller than the work function of ZrO_2_ (011), which is 5.363 eV. The E_vac_ of the CoAl‐LDH (012) and ZrO_2_ (011) were 0.881 and 2.751 eV, respectively. Their Fermi energy levels were −3.538 and −2.612 eV, which were obtained by the equations. The smaller work function of CoAl‐LDH compared to ZrO_2_ suggests charge transfer at their interface. The work function is a key parameter for manipulating the directional migration of electrons at heterogeneous interfaces. During formation of these interfaces, electrons transfer from the low component to the high Φ component. This electron transfer creates an IEF that optimizes the electronic structure of the Faraday reaction.^[^ ^51^, ^52^ ^]^ The large work function of ZrO_2_ will lead to the migration of electrons from CoAl‐LDH to ZrO_2_ when ZrO_2_ and CoAl‐LDH are combined until the Fermi energy level is balanced. Therefore, the part of CoAl‐LDH near the heterojunction contact interface loses electrons and is positively charged in order to achieve energy level balance, while the part of ZrO_2_ around the interface is negatively charged due to the electrons obtained, resulting in the energy band of CoAl‐LDH bending upward and the energy band of ZrO_2_ bending downward, forming the IEF from CoAl‐LDH to ZrO_2_. This is also consistent with XPS results. The density of states (DOS) spectrum is provided in Figure S6 (Supporting Information). The original ZrO_2_ has a distinct band gap around the Fermi level, presenting a regular electron distribution. However, the charge distribution of CoAl‐LDH is in a disordered state at the Fermi level, indicating that it has excellent electronic conductivity and rapid interfacial charge transfer ability. The distribution of electronic states near the Fermi level is denser, which helps to enhance its catalytic activity. DFT calculation shows that the effective S‐scheme heterojunction can be established through the combination of ZrO_2_ and CoAl‐LDH, which conforms to the charge transfer mechanism path of S‐scheme heterojunction.

### 3.6 Photocatalytic Performance

**Figure 9:**
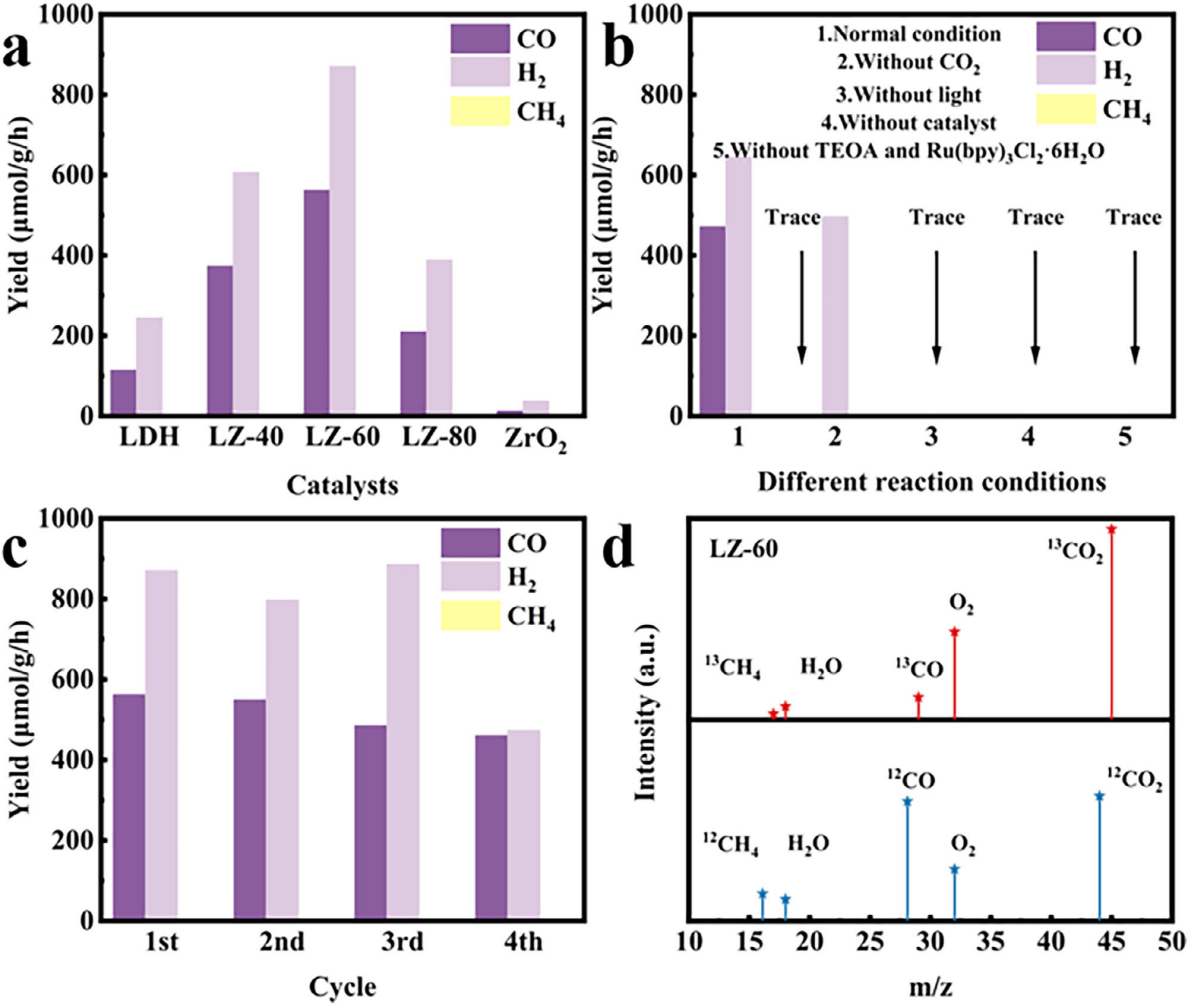
Yield of CO with a) different catalysts, b) different reaction conditions. c) Cycling tests for LZ‐60. d) The mass spectrum of mixture generated under ^13^CO_2_ and ^12^CO_2_ atmosphere.

As shown in **Figure** **9**a are the results of photocatalytic activity tests obtained under 1 h of light exposure with TEOA as the sacrificial agent, Ru(bpy)_3_Cl_2_·6H_2_O as the photosensitizer, and a xenon lamp (λ  =  320–760 nm) as the light source for the two original catalysts and composite catalysts with different ratios. The products obtained by photocatalysis in this reaction system are mainly CO and H_2_, while a very small amount of CH_4_ gas is also produced. From the Figure 9a, it can be seen that the CO yield of CoAl‐LDH is low at 114.378 µmol^−1^ g^−1^ h^−1^, and the CO yield of ZrO_2_ is extremely low at 13.214 µmol g^−1^ h^−1^. The CO yields of LZ‐40, LZ‐60, and LZ‐80 were 373.565, 562.545, and 210.138 µmol^−1^ g^−1^ h^−1^, respectively, which were significantly improved compared with those of the original catalysts, and the degree of improvement showed a tendency of increasing and then reducing. When the mass ratio of ZrO_2_ increased from LZ‐60 to LZ‐80, the photocatalytic performance started to decrease, which might be due to the fact that the excess ZrO_2_ would occupy the active site of CO_2_ photocatalysis and further agglomerate as the complex center of photogenerated e^−^‐h^+^ pairs, thus reducing the photocatalytic efficiency.^[^ ^38^ ^]^ The H_2_ yields of CoAl‐LDH and ZrO_2_ were 245.096 and 37.555 µmol^−1^ g^−1^ h^−1^, respectively, and those of LZ‐40, LZ‐60, and LZ‐80 were 606.684, 870.990, and 389.159 µmol^−1^ g^−1^ h^−1^, respectively, which showed the same growth as that of CO yield for the different proportions of catalysts trend. Very little CH_4_ gas was also present in the products, with CH_4_ yields of 0.866, 0.299, 0.118, 0.142, and 0.148 µmol^−1^ g^−1^ h^−1^ for CoAl‐LDH, LZ‐40, LZ‐60, LZ‐80, and ZrO_2_, respectively. The photocatalytic CO yield of the composite catalyst LZ‐60 with the optimal ratio was 5 times higher than that of single CoAl‐LDH and 43 times higher than that of single ZrO_2_. It can be seen that the heterojunction catalyst constructed by compositing CoAl‐LDH with ZrO_2_ has an excellent enhancement effect on CO production from photocatalytic reduction of CO_2_. Figure 9c shows that after four cycling experiments, LZ‐60 still maintains good photocatalytic activity for CO_2_ photoreduction, keeping 82% of the initial tested reduction performance. The catalyst loss during the experimental process and the inactivation of the active sites due to the surface adsorption of the reaction intermediates in the catalytic reduction stage were the main reasons for the slight decrease of the catalyst activity.^[^ ^53^ ^]^ Figure S7 (Supporting Information) shows the SEM image of the composite catalyst LZ‐60 after the cycling test, which is in good agreement with the morphology of the pristine LZ‐60, indicating that the constructed heterojunction composite catalyst has good structural stability. Shown in Figure 9b,d present a series of control experiments and isotopic labeling tests, which were conducted to exclude the interference from exogenous carbon‐containing organic species, TEOA, and photosensitizers, thereby ensuring that the detected CO product originates exclusively from CO_2_RR. The control experiments shown in Figure 9b are CO_2_ photocatalytic reduction experiments performed using pure argon (Ar) as the reaction gas, in a dark environment, with no catalyst, no TEOA, and no photosensitizer, respectively, compared with the products under normal conditions. The reaction in Ar shows a clear H_2_ precipitation reaction with traces of CO and CH_4_ (Group 2). This further supports the fact that CO was produced by a photocatalytic CO_2_RR on LZ‐60, which is consistent with the results of isotope labeling experiments. There was no photocatalytic activity on LZ‐60 under dark conditions, implying that the reaction was a photoreactive process (Group 3). Similarly, in the absence of a catalyst, there is almost no CO production during the photoreaction (Group 4). The absence of TEOA also does not lead to photocatalytic CO production (Group 5), which suggests that the sacrificial reagent plays a crucial role in the photocatalytic redox process. The results of five controlled experiments show that the CO product was obtained by light irradiation of the photocatalyst under a CO_2_ atmosphere, accompanied by the participation of sacrificial agents and photosensitizers, after CO_2_RR. Isotope analysis experiments using ^13^CO_2_ as the reaction gas further verified the product source. Figure 9d shows the mass spectrum, after irradiating LZ‐60 with a simulated light source (Xe lamp) in a ^13^CO_2_ atmosphere, where the clear peak at m/z = 29 was ^13^CO and m/z = 17 was entirely ^13^CH_4_. The combination of isotope analysis and comparative experiments reveals that the photogenerated electrons in LZ‐60 convert CO_2_ to CH_4_ and CO. The comparison of the photocatalytic activity and selectivity for the photocatalytic reduction of CO_2_ to CO with other studies is presented in Figure S8 and text Table S3 (Supporting Information), and LZ‐60 exhibits excellent catalytic efficiency.

### 3.7 Photoelectrochemical Properties Analysis

**Figure 10:**
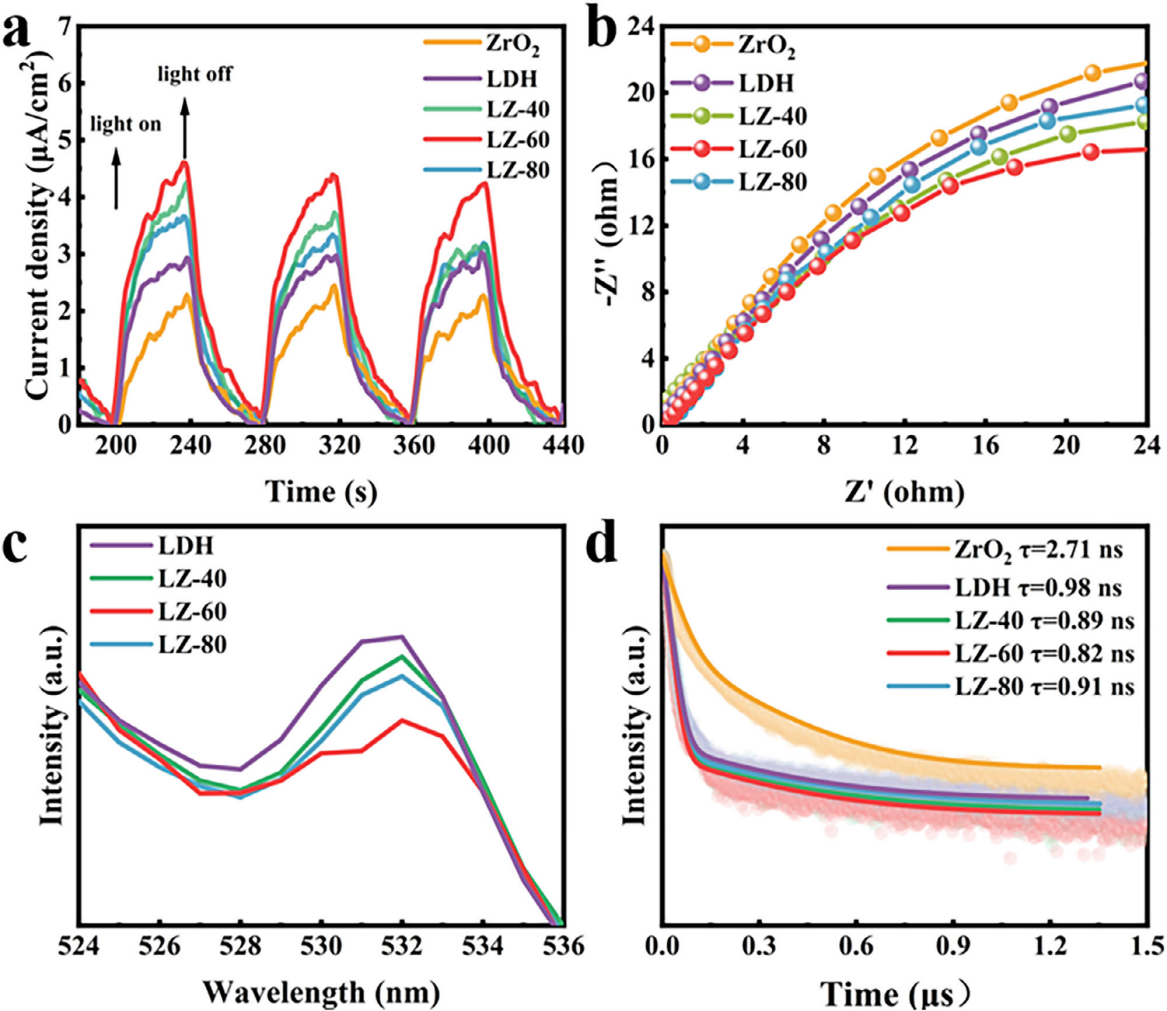
a) Transient photocurrent responses spectroscopy. b) EIS Nyquist plots. c) Normalized PL spectroscopy. d) Time‐resolved transient photoluminescence spectra.

The photogenerated charge transfer and separation efficiency of photocatalysts were probed by transient photocurrent profiles. The transient photocurrent response (TPR) test was performed on CoAl‐LDH, ZrO_2_, and LZ‐40, LZ‐60, and LZ‐80 by switching on and off the light at 40 s intervals, as shown in **Figure** **10**a. All the samples tested showed significant reversible photocurrent response under light or dark conditions, the order of photocurrent density was LZ‐60 > LZ‐40 > LZ‐80 > CoAl‐LDH > ZrO_2_, and the photocurrent of the composite heterojunction catalysts was larger than that of the pristine CoAl‐LDH and ZrO_2_, which indicated that the formed heterostructures could effectively promote the transfer of the photogenerated e^−^‐h^+^ pairs. The strong interaction between the two catalysts improved the aggregation of photogenerated carriers and accelerated the separation of e^−^‐h^+^ pairs. The highest photocurrent density of LZ‐60 indicates that the designed composite catalyst promotes the separation and directional motion of photogenerated carriers,^[^ ^54^, ^55^ ^]^ and the construction of S‐scheme heterojunction improves the photocatalytic activity. As shown in the electrochemical impedance spectra (EIS) of Figure 10b, ZrO_2_ presents the largest arcs, indicating that its interfacial resistance inhibits the transfer of photocarriers. The composite heterojunction catalyst presents smaller arcs in the Nyquist plot, indicating that the heterostructure has a lower charge migration resistance, which contributes to the rapid transport and separation of photogenerated carriers. Among the pristine catalysts and the composite heterojunction catalysts with different ratios, LZ‐60 presents the smallest arc with the smallest radius and the highest conductivity for charge transfer, indicating that the interfacial structure of the heterojunction catalysts shortens the charge transfer paths,^[^ ^56^ ^]^ facilitates the transport of charges, reduces the obstruction to the transfer of light carriers, and improves the charge transfer efficiency. Meanwhile, the electric field effect inside the heterojunction inhibits the electron‐hole pairs complex in LZ‐60 and promotes the separation of carriers, thus enhancing the photocatalytic efficiency. The photogenerated carrier transfer and separation efficiencies of CoAl‐LDH and LZ‐*x* were further investigated via photoluminescence (PL) spectroscopy (Figure 10c). The excitation wavelength is 485 nm. Low peak intensities were always associated with better photogenerated e^−^‐h^+^ pair separation efficiencies,^[^ ^47^ ^]^ and LZ‐60 had the lowest peak intensity among the tested samples, indicating its effective separation of photogenerated e^−^‐h^+^, which was attributed to the IEF force of the heterojunction to promote the transfer and separation of e^−^‐h^+^ pairs. The time‐resolved photoluminescence (TRPL) of two original catalysts and three composite catalysts under 480 nm optical excitation is shown in Figure 10d. The average lifetime was calculated by the equation $\tau =\frac{C_{1}{\tau_{1}}^{2}+C_{2}{\tau_{2}}^{2}}{C_{1}\tau_{1}+C_{2}\tau_{2}}$, where τ_1_ and τ_2_ represent the fluorescence emission lifetimes of distinct decay components, C_1_ and C_2_ denote their corresponding pre‐exponential amplitudes, and τ is the weighted average lifetime.^[^ ^57^ ^]^ The lifetime decay curves were fitted to obtain a new set of plots. The fitted curves give the average lifetime of CoAl‐LDH, ZrO_2_, LZ‐40, LZ‐60, and LZ‐80 as 0.98, 2.71, 0.89, 0.82, and 0.91 ns, respectively. Comparison of average photogenerated carrier lifetimes for all catalysts, ZrO_2_ has the highest and LZ‐60 has the lowest average lifetime, which suggests that the charge transfer efficiency inside the composite catalyst is increased, and the constructed S‐scheme heterojunction promotes the separation of photogenerated carriers. The observed reduction in fluorescence lifetime indicates that ZrO_2_ passes through a nonradiative electron‐hopping channel to CoAl‐LDH, thereby facilitating rapid interfacial transfer within the LZ‐*x* heterostructure. It can also be observed from the linear scanning volt‐ampere (LSV) curve (Figure S9, Supporting Information) that the heterojunction catalyst can significantly increase the cathode current density, and the combined CoAl‐LDH/ZrO_2_ is higher than that of the original ZrO_2_ and CoAl‐LDH. Among them, the LZ‐60 is the highest. This result well demonstrates that CoAl‐LDH/ZrO_2_ can achieve rapid surface kinetic reactions and effective photogenerated charge separation,^[^ ^58^ ^]^ which is consistent with other photoelectric and electrochemical experiments. Therefore, the analysis of the photoelectric test results of the pristine and composite catalysts indicates that the constructed heterojunction LZ‐*x* can effectively accelerate the generation, dissociation, and transport of photogenerated carriers, and increase the effective utilization of electrons in the photocatalytic reduction of CO_2_, thus improving the performance of CO_2_RR.

### 3.8 Photocatalytic CO_2_ Reduction Mechanism

**Figure 11:**
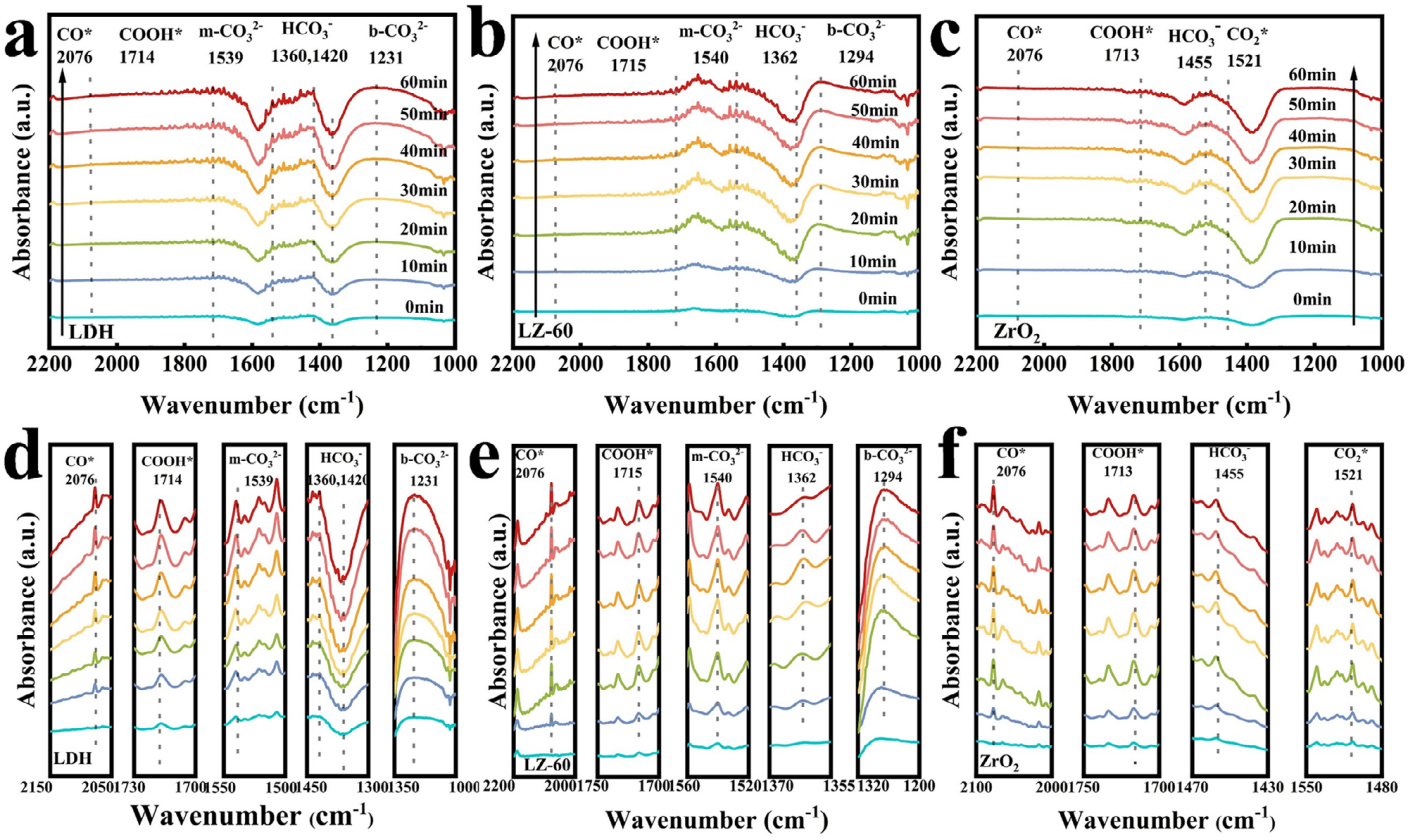
In situ FTIR spectra of a) CoAl‐LDH, b) LZ‐60, c) ZrO_2_. Magnified in situ FTIR spectra of d) CoAl‐LDH, e) LZ‐60, f) ZrO_2_.

The reaction intermediates of CoAl‐LDH, ZrO_2_, and LZ‐60 in the CO_2_RR process are characterized using in situ FTIR spectroscopy (**Figure** **11**a–f), respectively. The experimental equipment is shown in Figure S10 (Supporting Information). The test lasted 60 min. The initial 10 min were carried out in a dark environment, and H_2_O and CO_2_ were passed through at a gas flow rate of 10 mL min^−1^, while the reaction intermediates in the CO_2_ adsorption process were collected and characterized. After that, the Xe lamp was turned on for 50 min of spectral collection. With increasing irradiation time, the signal intensity of COOH*, a key intermediate of CoAl‐LDH, ZrO_2_, and LZ‐60, was gradually enhanced at 1714, 1715, and 1713 cm^−1^. COOH* was essential for the subsequent transformation into CO or CH_4_.^[^ ^59^ ^]^ CoAl‐LDH, ZrO_2,_ and LZ‐60 detected the signal of the intermediate CO* at 2076 cm^−1^, and CO* could eventually desorb to yield CO.^[^ ^60^ ^]^ The 1360 and 1420 cm^−1^ of CoAl‐LDH and the 1362 cm^−1^ of LZ‐60 are considered to be the symmetric stretching peaks of the bicarbonate (${\mathrm{HCO}}_{3}^{-}$) produced by the reaction of adsorbed H_2_O and CO_2_.^[^ ^61^ ^]^ The peaks of CoAl‐LDH and LZ‐60 at 1539, 1540 cm^−1^ represent the monodentate carbonate group (${m-\mathrm{CO}}_{3}^{2-}$), whereas the peaks at 1231, 1294 cm^−1^ show the bidentate carbonate group (${b-\mathrm{CO}}_{3}^{2-}$).^[^ ^61^ ^]^ In a comprehensive consideration based on the above results, under the simulated sunlight illumination CO_2_ is captured and adsorbed on the active sites of the catalysts, and the adsorbed CO_2_ accepts the electrons generated by the coupling of the CB of CoAl‐LDH with H^+^, aggregates COOH*, and then protonated to CO*, which may therefore desorb from the active site to produce CO,^[^ ^62^ ^]^ or be further protonated to CHO* and ultimately CH_4_,^[^ ^63^ ^]^ and it is clear that in this study, both CoAl‐LDH and LZ‐60 primarily fit the first scenario. Possible avenues can be proposed as follows:

(2) $$CO_{2}↑\to CO_{2}*$$

(3) $$CO_{2}*+e^{-}+H^{+}\to \mathrm{COOH}*$$

(4) $$\mathrm{COOH}*+e^{-}+H^{+}\to \mathrm{CO}*+H_{2}O$$

(5) CO∗→CO↑

(6) $$\mathrm{or}\;\mathrm{CO}*+e^{-}+H^{+}\to \mathrm{CHO}*$$

(7) $$\mathrm{CHO}*+e^{-}+H^{+}\to CH_{2}O*$$

(8) $$CH_{2}O*+e^{-}+H^{+}\to CH_{3}O*$$

(9) $$CH_{3}O*+e^{-}+H^{+}\to CH_{4}↑+O*$$

## 4 Conclusion

The S‐scheme heterojunction catalyst LZ‐60, which has a strong IEF, was created by combining CoAl‐LDH and ZrO_2_. The composite catalyst's CO_2_ photoreduction performance improved slightly compared to CoAl‐LDH and ZrO_2_. The highest yield of LZ‐60 after 1 h of photocatalytic reduction was 562.545 µmol^−1^ g^−1^ h^−1^, which is five times higher than single CoAl‐LDH and 43 times higher than single ZrO_2_. UV–vis and Mott‐Schottky spectra provide sufficient evidence for the construction of the heterojunction and the bandgap structure. The XPS characterization of CoAl‐LDH, ZrO_2_, and LZ‐60 indicates that electrons transfer from CoAl‐LDH to ZrO_2_ after combination, resulting in an IEF at the interface. The existence of internal electron transfer and IEF within the constructed LZ‐*x* composite heterojunction is further verified by DFT theoretical calculations. Meanwhile, the XPS characterization of LZ‐60 before and after the photocatalytic reaction was compared to verify the photogenerated electron transfer mechanisms in the catalysts. After the photocatalytic tests, the binding energies of the Co and Al elements decreased, while the opposite occurred for the Zr element. This indicates that ZrO_2_ electrons were transferred to the CoAl‐LDH under light. This result once again verifies that it conforms to the S‐scheme heterojunction charge transfer mode. A series of subsequent photoelectrochemical characterizations, such as PL and EIS tests, demonstrated that the S‐scheme heterojunction significantly enhanced electron transfer and separation. In situ FTIR characterization showed that CO* intermediate, rather than CHO*, dominated the main component in the photocatalytic CO_2_RR process of LZ‐60, which demonstrated its superior selectivity for CO. All the above provide a valuable reference method for designing an efficient CO_2_ photoreduction photocatalyst.

## Conflict of Interest

The authors declare no conflict of interest.

## Supporting information



Supporting Information

## Data Availability

The data that support the findings of this study are available on request from the corresponding author. The data are not publicly available due to privacy or ethical restrictions.
