# Supplementary material for: Strong Internal Electric Field‐Driven S‐scheme CoAl‐LDH/ZrO2 Heterojunction for Enhanced Photocatalytic CO2 Reduction: Configuration, Performance, and Mechanism
Source: Adv Sci (Weinh). 2025 Sep 12;12(45):e10939. doi: 10.1002/advs.202510939 (PMC12677695; doi:10.1002/advs.202510939)
Supplement: Supplementary file 1 — Supporting Information [file ADVS-12-e10939-s001.docx]

**Strong Internal Electric Field-Driven S-scheme CoAl-LDH/ZrO_2_ Heterojunction for Enhanced Photocatalytic CO_2_ Reduction: Configuration, Performance and Mechanism**

Mengwei Chen^a^, Jiaze Xiao^a^, Yongxin Lei^a^, Xupeng Qin^c^, Santosh K. Tiwari^d^, Nannan Wang^a^*, Zhiyao Wu^a,b^*, Xinpeng Wang^a^*, Yanqiu Zhu^a,e^*

^a^ State Key Laboratory of Featured Metal Materials and Life-cycle Safety for Composite Structures, Guangxi Key Laboratory of Processing for Non-Ferrous Metals and Featured Materials, School of Resources, Environment and Materials, Guangxi University, Nanning 530004, China.

^b^ State Key Laboratory of Chemistry for NBC Hazards Protection, Frontiers Science Center for Rare Isotopes, School of Nuclear Science and Technology, Lanzhou University, Lanzhou 730000, China.

^c^ National Synchrotron Radiation Laboratory, University of Science and Technology of China, Hefei 230029, China.

^d^ Department of Chemistry, NMAM Institute of Technology, Karnataka, India

^e^ Faculty of Environment, Science and Economy, University of Exeter, EX4 4QF, UK.

**Corresponding author.*

*wangnannan@gxu.edu.cn (Nannan Wang), wzhiyao2024@lzu.edu.cn (Zhiyao Wu), Xingpeng Wang@gxu.eud.cn (Xinpeng Wang), y.zhu@exeter.ac.uk (Yanqiu Zhu)*

**Text S1. Chemicals source**

Zirconyl chloride octahydrate (ZrOCl_2_·8H_2_O, 99%, Macklin), sodium hydroxide (NaOH, 99%, Guangdong Guanghua Science and Technology Co., Ltd.), urea (CO(NH_2_)_2_, 99%, Guangdong Guanghua Science and Technology Co., Ltd.), ammonium fluoride (NH_4_F, 99%, Guangdong Guanghua Science and Technology Co., Ltd.), cobalt nitrate (Co(NO_3_)_2_·6H_2_O, 99%, Guangdong Guanghua Science and Technology Co., Ltd.), aluminum nitrate (Al(NO_3_)_3_·9H_2_O, 99%, Guangdong Guanghua Science and Technology Co., Ltd.).

**Text S2. Characterizations**

The crystal structure was detected by the X-ray diffractometer (XRD, Rigaku D/MAX 2500 V, Rigaku Corporation, Japan) with the Cu Ka radiation (l = 0.15418 nm, 40 kV and 100 mA). The microstructure and lattice arrangements were investigated by scanning electronic microscopy (SEM, Sigma 300, Carl Zeiss, Germany) and transmission electronic microscopy (TEM, JEM-2100F, JEOL, Japan). The elemental distribution of the samples was detected by using energy dispersive spectroscopy (EDS) of Oxford Instrument. The elemental microanalysis and atom binding states were examined by X-ray photoelectron spectroscope (XPS, ESCALAB 250XI, Waltham, USA) with an Al Ka radiator. These XPS spectrum were corrected by using C 1s spectra with the calibration energy of 284.8 eV. The infrared spectra further provided information of surface functional groups and chemical bonds through a Fourier transform infrared spectrometer (FT-IR, Nicolet iS50, Thermo Fisher Scientific, USA). The photoelectric performance of as prepared samples was estimated using an electrochemical workstation (CHI660D, Chenhua nstrument, China) with a three-electrode system. Pt and Ag/AgCl electrodes were selected as the counter and reference electrodes, while catalyst coated on pure uorine-doped tin oxide (FTO) glasses (1 * 1 cm^2^ acted as working electrodes, with 0.5 M Na_2_SO_4_ as the electrolyte solution. Transient photocurrent responses spectroscopy, EIS and Mott-Schottky curves were tested under the open circuit voltage and Mott-Schottky curves were tested using 500,1000,1500 Hz and performed two times to reduce errors. Linear scanning voltammetry (LSV) testing is carried out with a scanning rate of 100 mV/s. A full spectrum Xe lamp (300 W, LS-SXE300, Perfect light, China) was adopted as the light source deposited 10 cm away from the testing system to realize the transient photocurrent responses spectroscopy experiment. UV-vis diffuse reflectance spectra (DRS, UV-3600Plus, SHIMADZU, Japan) were applied to investigate the light-harvesting ability of the catalysts. Photoluminescence spectra (PL, FL3C-111 TCSPC, HORIBA, Japan) was obtained to study the recombination degree of photoinduced electrons and holes upon different samples. Co K-edge and Zr K-edge XAFS is measured in the HXMA and SXRMB beamlines of Canadian Light Source (CLS). These data are collected at room temperature under air atmosphere. Soft XAS measurements were carried out at spherical grating monochromator (SGM) beamline at Canadian Light Source under vacuum condition. N_2_ adsorption-desorption isotherms (Autosorb iQ-C-MP, Quantachrome Instruments, USA) were measured to test investigate the BET surface areas. *In-situ* FTIR was used to analyze the reaction intermediates during CO_2_RR. Each sample was firstly purged with high purity Ar gas (99.999%) for 30 min at room temperature to discharge interference and collect the background, then high purity CO_2_ (99.999%) with water vapor was introduced with the flow rate of 10 mL/min and all spectral data were recorded. After 10 min CO_2_ adsorption, 300 W Xenon lamp (PLS-FX300HU, Perfectlight, China) was turned on and the background measured by subtracting pure Ar gas from the spectrum obtained from the test was the catalyst test data.

**Text S3.** **DFT calculations.**

All DFT calculations were performed using the Vienna Ab initio Simulation Package (VASP) with the GGA-PBE functional valence electrons are considered with a kinetic energy cutoff of 500 eV. The convergence criteria for the self-consistency of the electron energy and the geometric optimization of the force are severally 10^-6^ eV/atom and 0.02 eV/Å. The Brillouin zone is sampled employing the MonkhorstePack method and integrated with all structures of the Monkhorst-Pack 1 × 1 × 1 k-point network. A 15 Å vacuum layer and dipole corrections prevented spurious interactions. Work functions were calculated as $\text{Φ=}\text{E}_{\text{vacuum}}\text{-}\text{E}_{\text{Fermi}}$.

**Text S4.** **Catalytic performance test.**

10 mg of photocatalyst was dispersed uniformly in a quartz reactor with the triethanolamine (TEOA), acetonitrile and DI water inside, which adhered to a ratio of 1:1:3, further 3 mg Ru(bpy)3Cl_2_·6H_2_O was added as the photosensitizer. A 300 W Xe arc lamp (PLS-SXE300D, Beijing Perfectlight), used as the UV-vis light source (320 nm < l < 780 nm), was placed at a distance about 10 cm from the reactor. Before photocatalytic test, the reactor was pumped with pure CO_2_ for 30 min under the flow rate of 100 ml/min, further illuminating for 1 h to test the CO_2_RR performance. During the photocatalytic test, the ambient temperature was kept around 25℃ through a cooling water external connection system. After each photocatalytic test, the used sample was washed with DI water and ethanol for several times through vacuum filtration, and dried at 60 C for 12 h at vacuum drying oven to get activity recover, this procedure was carried out for four times to measure the stability of catalysts. After test, gas products were collected using a peristaltic pump and detected by a gas chromatograph. Isotopic labeling test with the only input gas of ^13^CO_2_ was performed to determine the carbon source in the products by an assembly of mass spectrum and gas chromatography. The formulation of gaseous yield is listed as following:

Yield = $\frac{\text{C×V}}{\text{22.4×m×h}}$ mol·h^−1^·g^−1^

Where C represents the gas concentration (ppm), V represents the volume of reactor (mL), m represents the dosage of catalysts (mg) and h represents the irradiation time (hours). Productivity of gaseous products was calculated using aforesaid formula, and has been added in supporting information section.


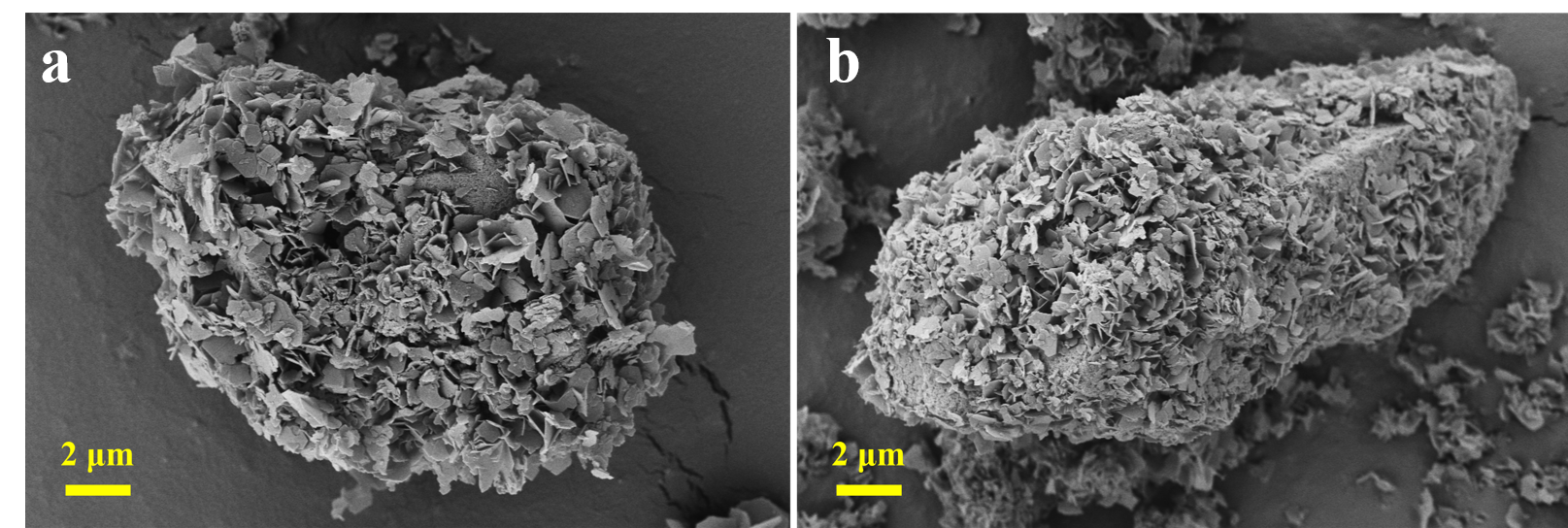


**Figure S1. SEM of (a) LZ-40 and (b) LZ-80**


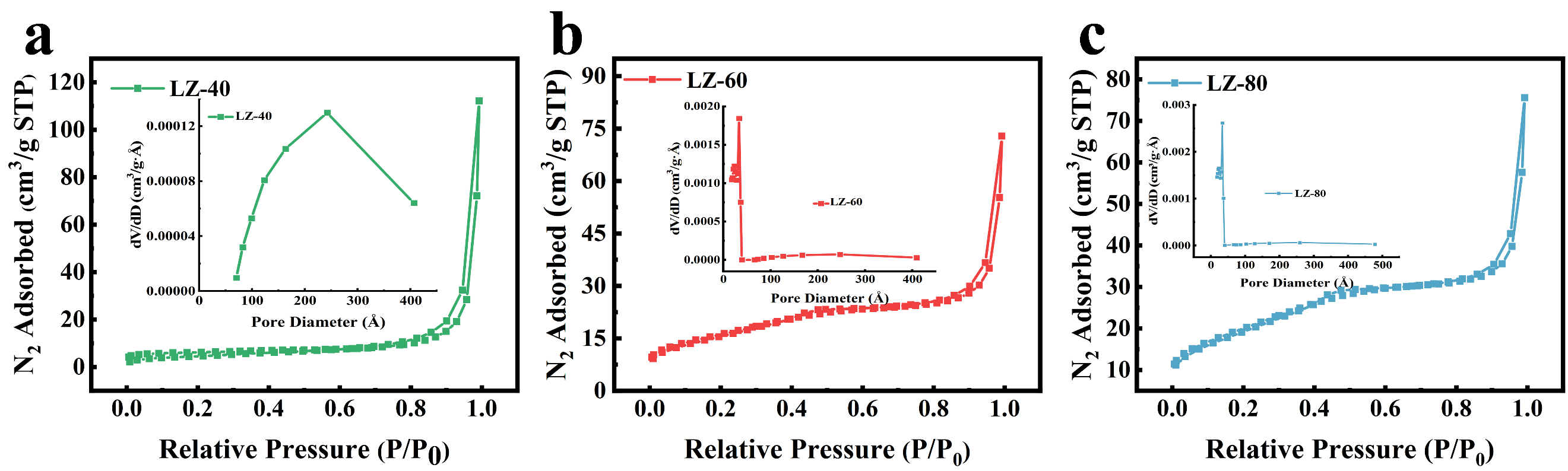


**Figure S2. Specific surface area and physical adsorption ability analysis. Related to Figure 3. N_2_ adsorption-desorption isotherms of (a) LZ-40 and (b) LZ-60, (c)LZ-80.**

**
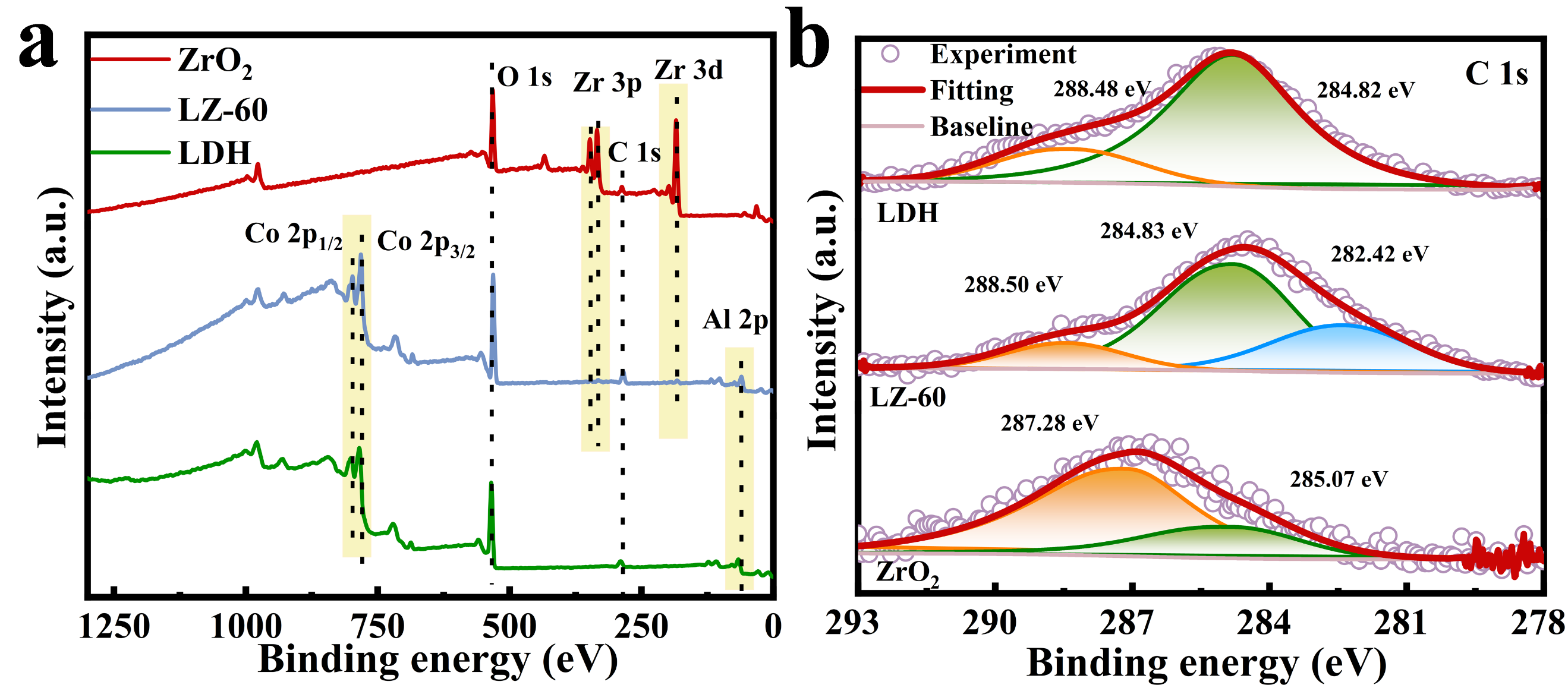
**

**Fig S3. XPS spectrum of (a) survey spectrum,** **(b) C 1s.**

**
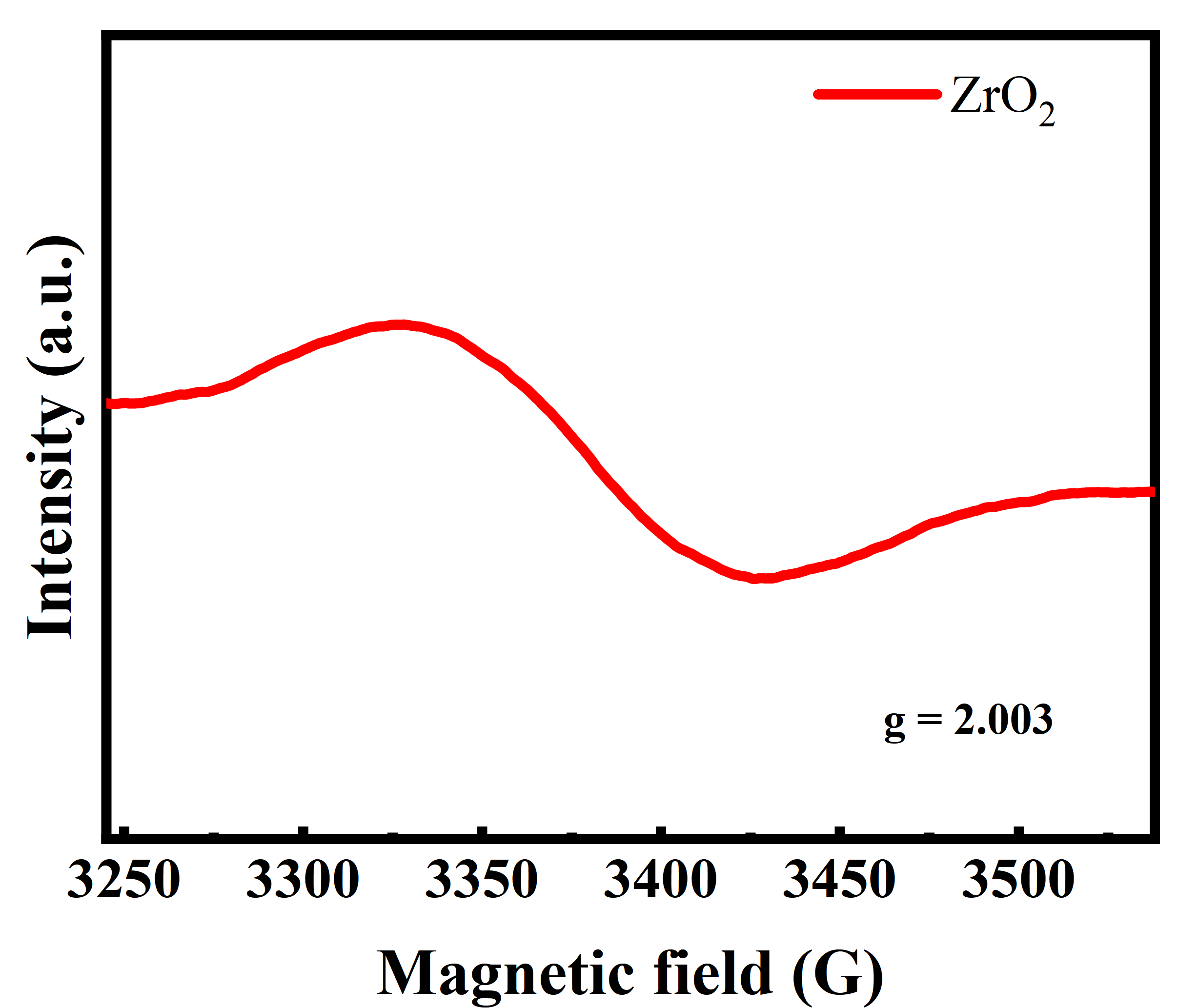
**

**Fig S4. EPR characterization of ZrO_2_.**

**
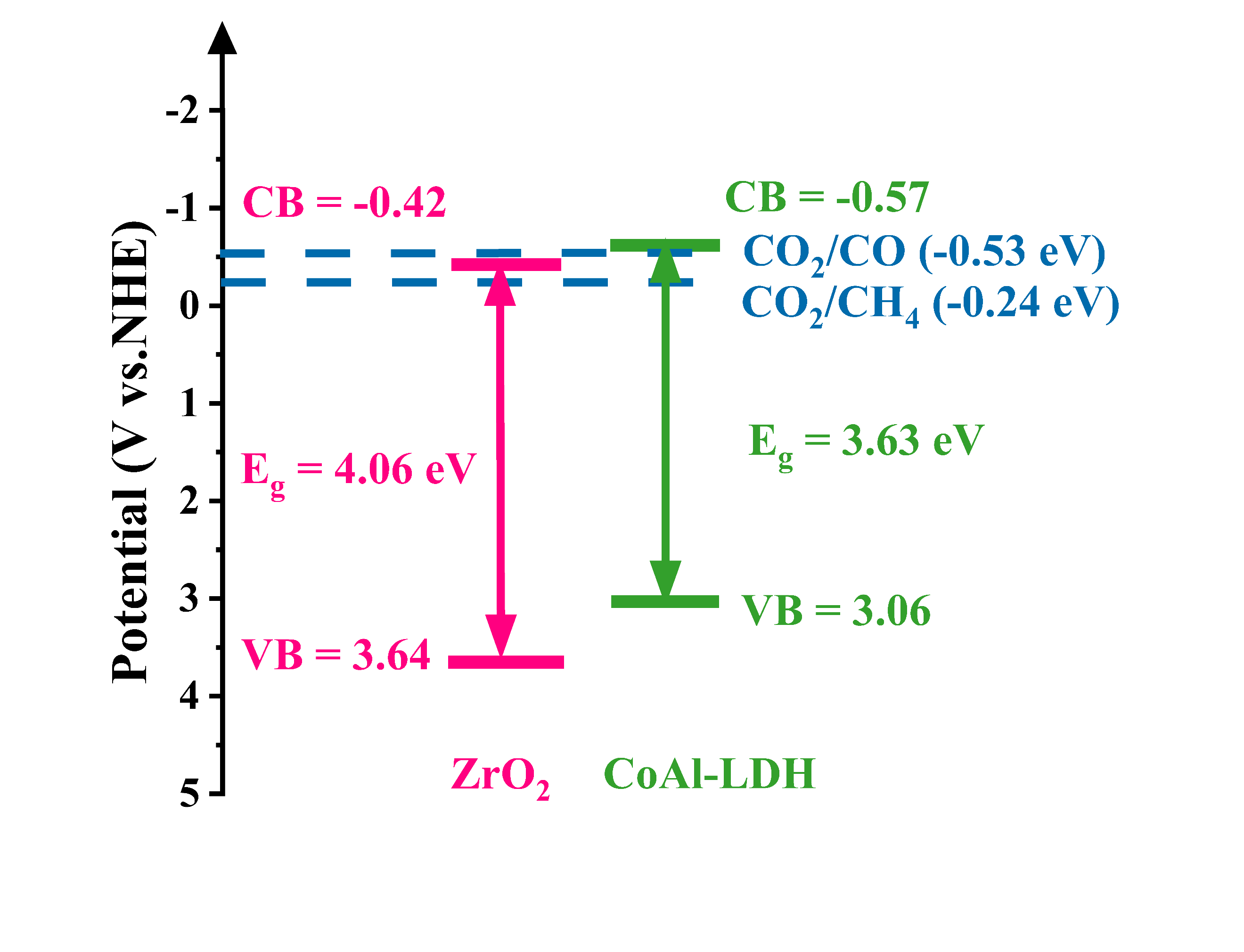
**

**Fig S5.** **The band structure of ZrO_2_ and CoAl-LDH.**

**
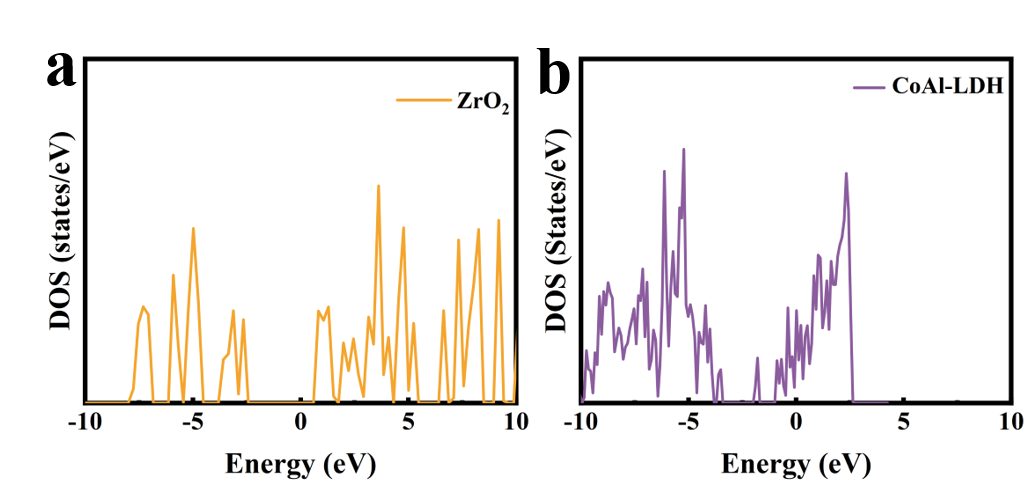
**

**Fig S6. The density of states (DOS) spectrum of ZrO_2_ and CoAl-LDH.**


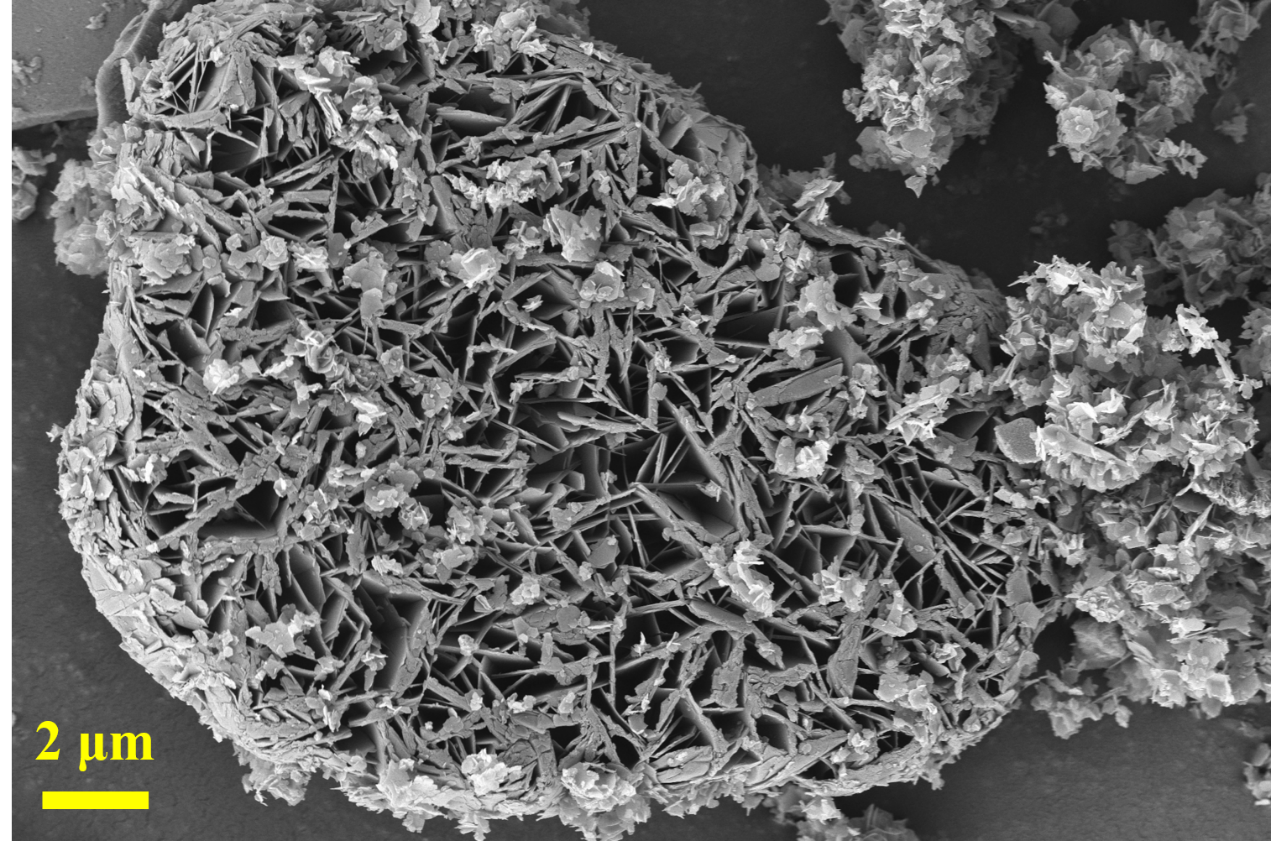


**Fig S7. SEM images of used LZ-60.**

**
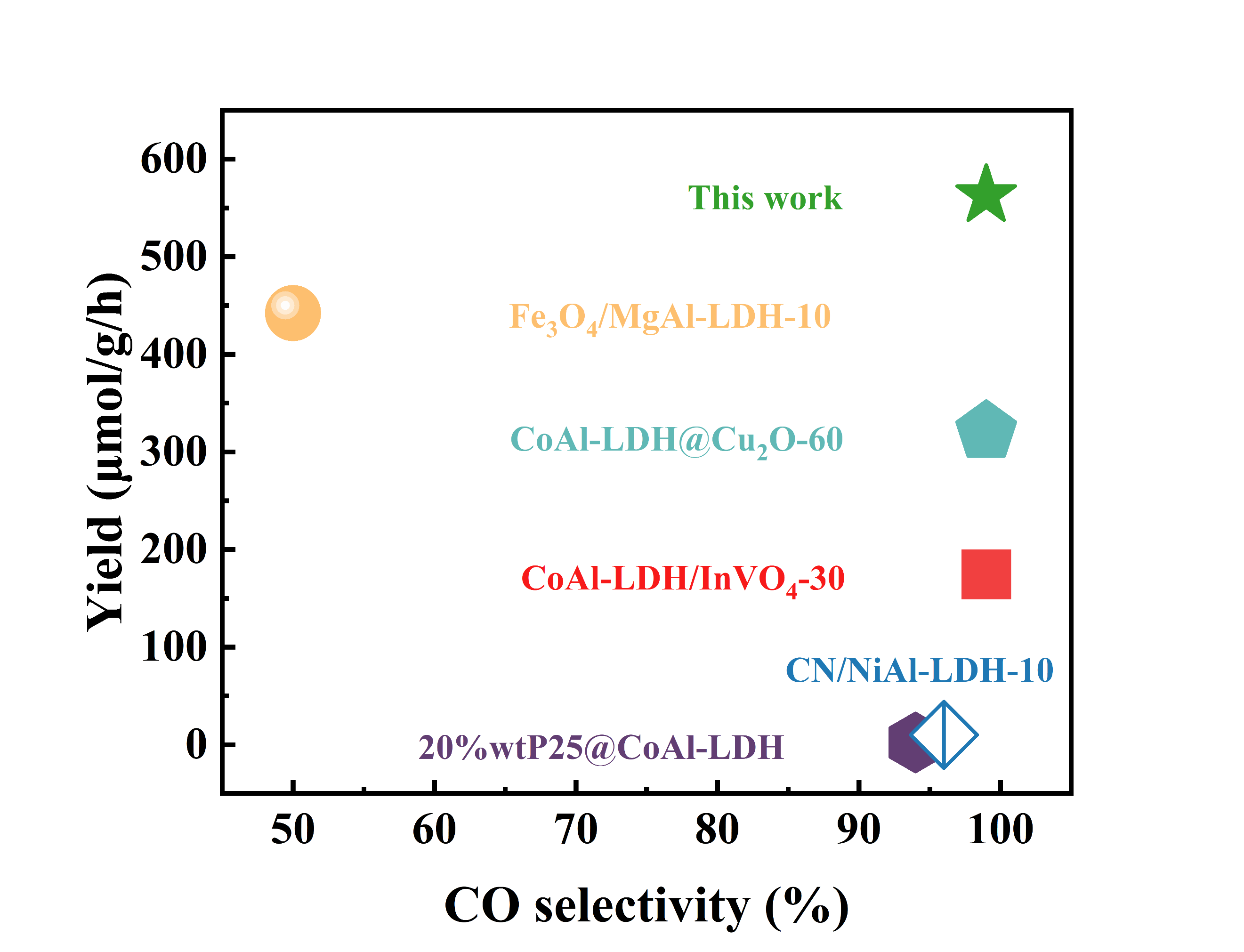
**

**Fig S8.** **Comparison of photocatalytic activity and selectivity for CO production with other works.**

**
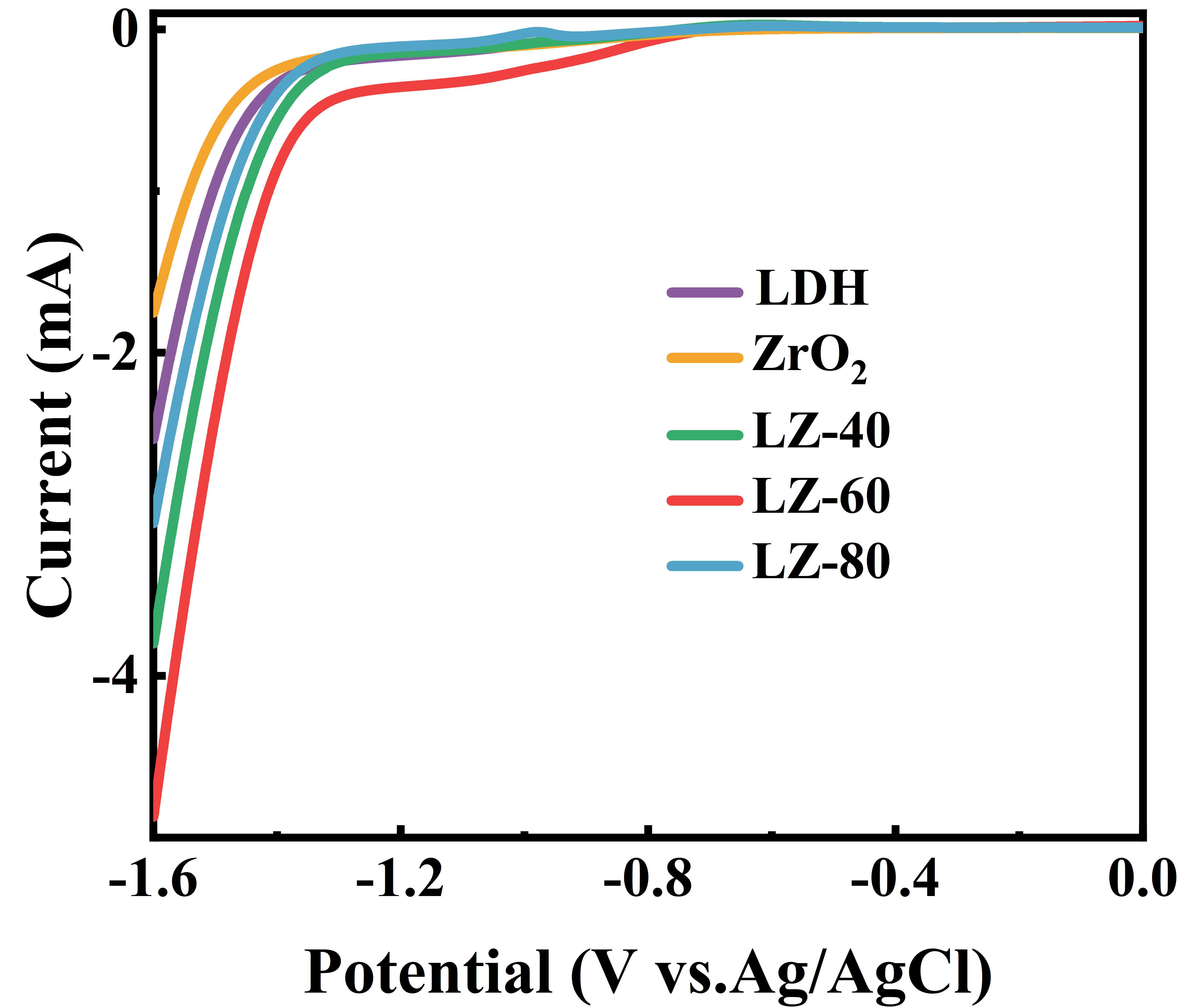
**

**Fig S9. LSV curves of various catalysts.**

**
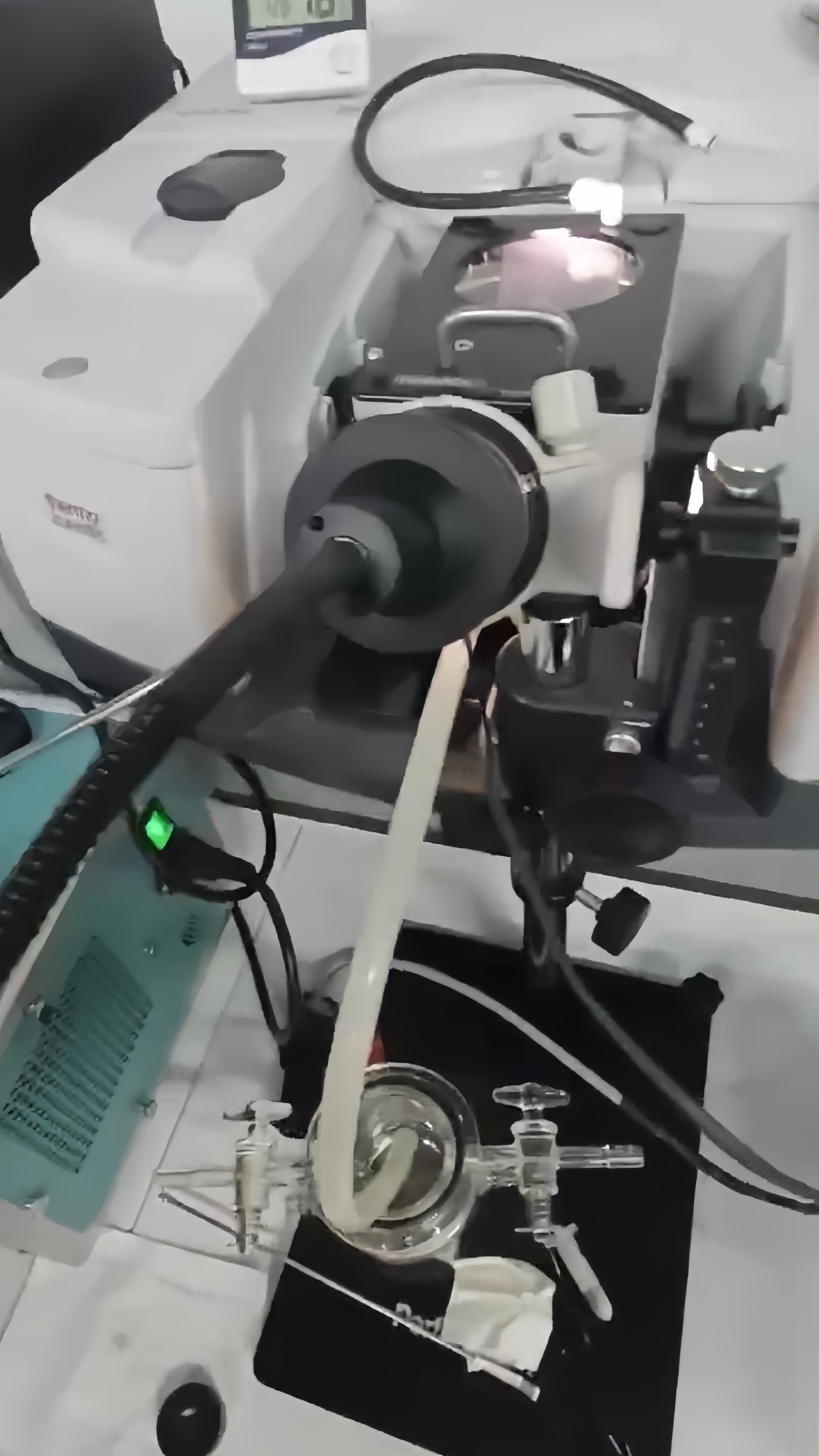
**

**Fig S10. *In situ* FTIR equipment.**

**Table S1. Elemental content of LZ-60. Correlates with the EDS diagram.**

| **Element** | **Wt%** | **Atomic%** |
| --- | --- | --- |
| O | 35.05 | 55.01 |
| Al | 2.18 | 2.03 |
| Co | 11.91 | 5.08 |
| Zr | 37.70 | 10.38 |

**Table S2. Specific surface area, pore volume and pore size of ZrO_2_, CoAl-LDH, LZ-40, LZ-60, and LZ-80**

| **Samples** | **SBET (m^2^/g)** | **Pore volume (cm^3^/g)** | **Average pore size(nm)** |
| --- | --- | --- | --- |
| **ZrO_2_** | 179.7079 | 0.1674 | 3.3006 |
| **LDH** | 19.8915 | 0.1412 | 36.5472 |
| **LZ-40** | 23.1446 | 0.1734 | 33.7527 |
| **LZ-60** | 57.0356 | 0.1126 | 8.8414 |
| **LZ-80** | 68.9505 | 0.1169 | 7.1291 |

**Table S3**. **Comparison of CO_2_ photoreduction performance of different photocatalysts.**

| **Catalyst** | **Photosensitizer** | **Sacrificial**  **agent** | **Condition** | **Evolution**  **(μmol/h/g)** | **Ref.** |
| --- | --- | --- | --- | --- | --- |
| CoAl-LDH/ZrO_2_ | Ru(bpy)_3_^2+^ | TEOA | 300 W Xe lamp | CO: 562.5  CH_4_: 0.1 | This work |
| P25@CoAl-LDH | / | / | 300 W Xe | CO=2.21 | [1] |
| CoAl-LDH@Cu_2_O | Ru(bpy)_3_^2+^ | TEOA | 300 W Xe lamp | CO: 320.9  CH_4_: 2.7 | [2] |
| CoAl-LDH/InVO_4_ | Ru(bpy)_3_^2+^ | TEOA | 300 W Xe lamp λ ＞  420 nm | CO: 87.2 | [3] |
| Fe_3_O_4_/MgAl-LDH | / | / | Ultraviolet light  (254 nm,8W) | CO: 442.2  CH_4_: 223.9 | [4] |
| R-ZnO@LDH | / | / | Xe (λ = 320-780nm) | CH_4_: 73.92 | [5] |
| MgAl-LDO/TiO_2_ | / | / | 450 W Xe | CO:1.5 | [6] |
| CoZnAl-LDH/RGO/g-C_3_N_4_ | / | / | 300 W Xe | CO:10.11 | [7] |
| g-C_3_N_4_/NiAl-LDH | / | / | 300 W Xe | CO:8.2 | [8] |
| CN/NiFe-LDH | / | / | 300 W Xe | CO:55.1 | [9] |
| HT150MgAlTi- LDH | / | / | 400 W  UV (200-1000 nm) | CO: 10 | [10] |

**Reference**

[1] S. Kumar, M.A. Isaacs, R. Trofimovaite, L. Durndell, C.M. Parlett, R.E. Douthwaite, B. Coulson, M.C. Cockett, K. Wilson, A.F. Lee, P25@ CoAl layered double hydroxide heterojunction nanocomposites for CO_2_ photocatalytic reduction, Applied Catalysis B: Environmental, 209 (2017) 394-404.

[2] Z. Wu, X. Wang, S. Deng, X. Qin, Q. Han, Y. Zhou, Y. Zhu, N. Wang, C. He, Y.A. Wu, Photocatalytic CO_2_ reduction of 2D/0D CoAl-LDH@ Cu_2_O catalyst with pn heterojunction, Iscience, 26 (2023).

[3] J. Wei, S. Zhang, J. Sun, T. Liang, Z. Li, Z. Li, X. Yi, R. Xiong, J. Deng, Z. Yu, Z-scheme CoAl-layered double hydroxide/indium vanadate heterojunction for enhanced and highly selective photocatalytic reduction of carbon dioxide to carbon monoxide, Journal of Colloid and Interface Science, 629 (2023) 92-102.

[4] G. Gao, Z. Zhu, J. Zheng, Z. Liu, Q. Wang, Y. Yan, Ultrathin magnetic Mg-Al LDH photocatalyst for enhanced CO2 reduction: Fabrication and mechanism, Journal of colloid and interface science, 555 (2019) 1-10.

[5] Q. Guo, Q. Zhang, H. Wang, Z. Liu, Z. Zhao, Core-shell structured ZnO@ Cu-Zn–Al layered double hydroxides with enhanced photocatalytic efficiency for CO_2_ reduction, Catalysis Communications, 77 (2016) 118-122.

[6] C. Zhao, L. Liu, G. Rao, H. Zhao, L. Wang, J. Xu, Y. Li, Synthesis of novel MgAl layered double oxide grafted TiO_2_ cuboids and their photocatalytic activity on CO_2_ reduction with water vapor, Catalysis Science & Technology, 5 (2015) 3288-3295.

[7] Y. Yang, J. Wu, T. Xiao, Z. Tang, J. Shen, H. Li, Y. Zhou, Z. Zou, Urchin-like hierarchical CoZnAl-LDH/RGO/g-C_3_N_4_ hybrid as a Z-scheme photocatalyst for efficient and selective CO_2_ reduction, Applied Catalysis B: Environmental, 255 (2019) 117771.

[8] S. Tonda, S. Kumar, M. Bhardwaj, P. Yadav, S. Ogale, g-C_3_N_4_/NiAl-LDH 2D/2D hybrid heterojunction for high-performance photocatalytic reduction of CO_2_ into renewable fuels, ACS Applied Materials & Interfaces, 10 (2018) 2667-2678.

[9] B. Zhu, Q. Xu, X. Bao, H. Yin, Y. Qin, X.-C. Shen, Highly selective CO_2_ capture and photoreduction over porous carbon nitride foams/LDH monolith, Chemical Engineering Journal, 429 (2022) 132284.

[10] H. Zhao, J. Xu, L. Liu, G. Rao, C. Zhao, Y. Li, CO_2_ photoreduction with water vapor by Ti-embedded MgAl layered double hydroxides, Journal of CO2 Utilization, 15 (2016) 15-23.
